# Disruption of CD47-SIRPα signaling restores inflammatory function in tumor-associated myeloid-derived suppressor cells

**DOI:** 10.1016/j.isci.2024.109546

**Published:** 2024-03-20

**Authors:** Carlo Zimarino, William Moody, Sarah E. Davidson, Hafsa Munir, Jacqueline D. Shields

**Affiliations:** 1 MRC Cancer Unit, Hutchison/MRC Research Centre, University of Cambridge, Cambridge, UK; 2 The Kennedy Institute of Rheumatology, University of Oxford, Oxford, UK; 3 Helmholtz Institute for Translational Oncology Mainz (HI-TRON Mainz) – A Helmholtz Institute of the DKFZ, Mainz, Germany; 4 German Cancer Research Centre (DKFZ), Division of Dermal Oncoimmunology, Heidelberg, Germany; 5 Comprehensive Cancer Centre, Kings College London, London, UK; 6 Centre for Cancer Sciences, School of Medicine, Biodiscovery Institute, University of Nottingham, Nottingham, UK

**Keywords:** Molecular biology, Immunology, Cell biology, Stem cells research, Cancer

## Abstract

Myeloid-derived suppressor cells (MDSCs) are a heterogeneous immune population with diverse immunosuppressive functions in solid tumors. Here, we explored the role of the tumor microenvironment in regulating MDSC differentiation and immunosuppressive properties via signal-regulatory protein alpha (SIRPα)/CD47 signaling. In a murine melanoma model, we observed progressive increases in monocytic MDSCs and monocyte-derived dendritic cells that exhibited potent T cell-suppressive capabilities. These adaptations could be recapitulated *in vitro* by exposing hematopoietic stem cells to tumor-derived factors. Engagement of CD47 with SIRPα on myeloid cells reduced their phagocytic capability, enhanced expression of immune checkpoints, increased reactive oxygen species production, and suppressed T cell proliferation. Perturbation of SIRPα signaling restored phagocytosis and antigen presentation by MDSCs, which was accompanied by renewed T cell activity and delayed tumor growth in multiple solid cancers. These data highlight that therapeutically targeting myeloid functions in combination with immune checkpoint inhibitors could enhance anti-tumor immunity.

## Highlights

• CD47-SIRPα signaling in MDSCs drives suppression of T cell proliferation in tumors
• Engagement of SIRPα reduces phagocytosis and antigen presentation by MDSCs
• Blocking SIRPα restores phagocytosis by MDSCs and enhances T cell activity
• Disruption of CD47-SIRPα signaling delays tumor growth

## Graphical abstract

**Figure:**
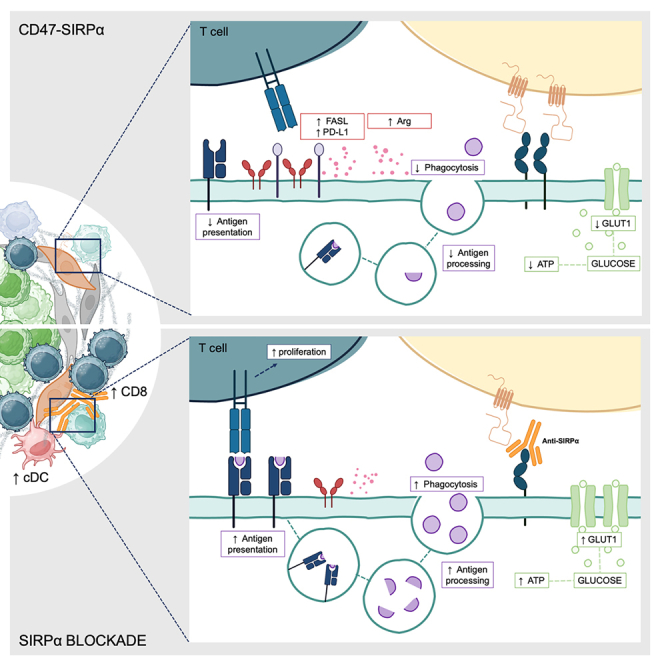

## Introduction

The advent of immune check point inhibitor (ICI) therapies that target receptors on T cells, such as CTLA4 and PD1, to reinvigorate anti-tumor immune responses has changed the landscape of cancer therapy. However, the magnitude and durability of responses vary considerably among patients and tumor types,^1^^,^^2^^,^^3^^,^^4^^,^^5^^,^^6^^,^^7^ with many patients developing therapeutic resistance and experiencing off-target toxicity.^8^^,^^9^ While T cells are key drivers of anti-tumor immunity and are as such a desirable target for therapeutic intervention, there are a multitude of other infiltrating immune populations within the tumor that play a critical role in tumor progression.

The tumor ecosystem is extremely diverse, rich in myeloid populations including monocytes, macrophages, neutrophils, and, importantly, dendritic cells (DCs).^10^^,^^11^ These innate immune constituents display potent immune modulatory properties through expression of immune regulatory molecules and by presenting antigen. Thus, targeting features of innate immune regulation represents an attractive alternative or synergistic opportunity to improve immune-based cancer therapies.

Tumor-infiltrating myeloid populations initially display anti-tumor functions, phagocytosing dying tumor cells and presenting antigen to T cells in the draining lymph node. However, as tumors grow, these functions are suppressed, and the cells acquire a pro-tumor phenotype.^11^^,^^12^^,^^13^^,^^14^^,^^15^^,^^16^^,^^17^ During tumorigenesis, emergency myelopoiesis gives rise to a heterogeneous population of myeloid cells termed myeloid-derived suppressor cells (MDSCs). On the molecular level, MDSCs are distinct from mature myeloid cells as they express key immunosuppressive pathways.^18^^,^^19^^,^^20^^,^^21^^,^^22^^,^^23^^,^^24^ Much like mature myeloid cells, murine MDSCs can be broadly identified based on expression of common myeloid markers, namely, CD11b and CD11c (which generally distinguish monocytic cells from DCs), as well as Ly6G and Ly6C (which can broadly differentiate monocytes, macrophages, and neutrophils).^25^^,^^26^ These markers distinguish the two main MDSC subtypes, CD11c^−^CD11b^+^Ly6G^+^ granulocytic MDSCs (G-MDSCs) and CD11c^−^CD11b^+^Ly6C^+^ monocytic MDSCs (M-MDSCs). While these markers are also commonly expressed by mature myeloid cells, MDSCs are distinguishable because they also exhibit potent T cell-suppressive capabilities and, in general, are associated with immune-suppressive behavior. Recent single-cell sequencing data indicate substantial overlap between the gene signatures of G-MDSCs and M-MDSCs;^27^ however, G-MDSCs are thought to play a critical role in antigen-specific reactive oxygen species (ROS)-driven T cell suppression^28^ and are prevalent in tumors of prostate and breast. In contrast, M-MDSCs, which preferentially accumulate in melanoma, are thought to elicit antigen-independent effects via release of nitric oxide and arginase, and production of immune-suppressive cytokines. DCs also play a role in tumor progression.^29^ Briefly, conventional dendritic cells 1 (cDC1) can activate cytotoxic T cells,^30^ conventional dendritic cells 2 (cDC2) are key inducers of anti-tumor CD4 T cell responses,^31^ and monocyte-derived DCs (moDCs) can efficiently induce T_reg_ activation and expansion.^32^^,^^33^ Therefore, in melanoma, M-MDSCs and moDCs are key contributors to the immune-suppressive microenvironment within the growing tumor.

Previous reports have shown that soluble mediators and receptor-antigen interactions within the tumor microenvironment (TME) can skew myeloid functionality to become pro- or anti-tumorigenic depending on the type and stage of disease. Importantly, CD47 expressed by tumor cells has been implicated in the switch in the development and function of the myeloid compartment within tumors. Additionally, CD47 expression by the tumor has been associated with poor prognosis.^34^^,^^35^ Previous reports have shown that CD47 binds to the immune checkpoint, signal-regulatory protein α (SIRPα or CD172a), which is expressed predominantly by myeloid cells. Engagement of SIRPα with CD47 inhibits phagocytosis by myeloid cells. Activation of this pathway has previously been shown to contribute to immune evasion in cancer by limiting clearance of tumor cells by macrophages.^36^^,^^37^ Engagement of the SIRPα pathway on myeloid populations through CD47 induces phosphorylation of immunoreceptor tyrosine-based inhibition motifs (ITIMs) cytoplasmic domain and activation of the SH2-containing tyrosine phosphatase (SHP-1/2) which in turn mediate an array of inhibitory functions.^38^^,^^39^ In addition to impairment of phagocytosis, CD47-SIRPα signaling within tumors has also been shown to inhibit DC maturation, antigen presentation, maintenance of MDSC function, and prevention of neutrophil migration.^40^^,^^41^

These observations have led to the development of agents targeting CD47. Its neutralization has been shown to reduce tumor growth and promote anti-tumor immunity in preclinical tumor models.^35^^,^^42^^,^^43^^,^^44^ The safety and efficacy of these agents are now being tested in clinical trials.^45^^,^^46^ Reports have shown that CD47 blockade mediates tumor resolution primarily through increased macrophage clearance^47^^,^^48^^,^^49^ and via enhanced antigen cross-presentation by DCs which leads to improved T cell priming.^43^^,^^47^ However, CD47 is ubiquitously expressed in healthy as well as diseased tissue, from tumor cells to erythrocytes, bladder, prostate, fallopian tubes, mediumly in bronchus tissue, salivary glands, and sex organs. Thus, its inhibition risks disrupting the function of normal, non-malignant cells, leading to unwanted clinical effects and toxicities such as the induction of anemia.^50^^,^^51^^,^^52^ Indeed, clinical trials, including Arch Oncology’s phase I/II clinical trial of Ti-061 and Celgene’s CC-90002, have been discontinued due to potential toxicity caused by anti-CD47 antibody therapy.

Targeting of SIRPα, the binding partner of CD47, may therefore offer a safer alternative approach with which to modulate myeloid-driven anti-tumor immunity, as it is almost exclusively expressed by the myeloid compartment.^53^ In support of this, several studies have alluded to enhanced myeloid cell activation post-SIRPα blockade. Indeed, Kuo et al. showed that treatment of mice bearing colon cancer with a combination of anti-PD-1/PD-L1 and a chimeric anti-SIRPα neutralizing antibody was highly effective in suppressing growth of primary tumors.^54^ In this model, anti-SIRPα treatment facilitated monocyte and DC activation and enhanced T cell effector functions. Other studies reported enhanced T cell infiltration post-treatment which overcame immunotherapy-induced immune exclusion.^53^ Furthermore, Zhao et al. showed that blockade of CD47 to interrupt this signaling pathway resulted in enhanced antibody-dependent cellular cytotoxicity of Her2/Neu breast cancer cells by neutrophils after trastuzumab treatment.^40^ However, this combination therapy had little efficacy in more aggressive tumor models, such as the B16.F10 melanoma model. Other studies have also highlighted the complexity of SIRPα signaling in the TME and how it may limit therapeutic efficacy although the mechanism is still unclear. Zhou et al. recently showed that mice bearing SIRPα-deficient melanomas had no response to anti-PD-L1 treatment, but SIRPα overexpression significantly enhanced immunotherapy response.^55^ The discrepancy between colon cancer and melanoma is fascinating and raises several key questions about the role of SIRPα in different tumor contexts and the potential contribution it makes at different stages of tumor evolution.

While T cell immune checkpoints have been well characterized, our understanding of the CD47-SIRPα axis across myeloid populations is less clear. Here, we explore the role of the CD47-SIRPα interactions on myeloid function in a B16.F10 melanoma model. We observed that the myeloid compartment of established tumor primarily consisted of M-MDSCs and moDCs. We confirmed widespread expression of CD47 within the TME while SIRPα expression was limited to myeloid cells. In contrast to Kuo et al., we show that disruption of CD47-SIRPα signaling following selective SIRPα blockade induces energetic rewiring of myeloid cells and is sufficient to slow tumor growth. This is mediated by restoration of phagocytosis, antigen processing and presentation, and a shift in CD8:Treg ratios. The effects of SIRPα blockade were not limited to melanoma and translated to other solid tumors, namely pancreatic and breast tumors showing a conserved anti-phagocytic pathway activated in suppressive myeloid cells across multiple tumor types. Our data indicate that approaches targeting phagocytosis pathways in myeloid populations, boosting antigen uptake and presentation to infiltrating T cells, may synergize with conventional immune checkpoint inhibitors and enhance anti-tumor immunity. Therapeutically targeting both innate and adaptive arms may enable the use of lower doses to lower toxicities currently experienced by patients undergoing treatment with T cell-targeted immune checkpoint inhibitors or anti-CD47 agents.

## Results

### The myeloid compartment dynamically evolves during tumor development

**Figure 1:**
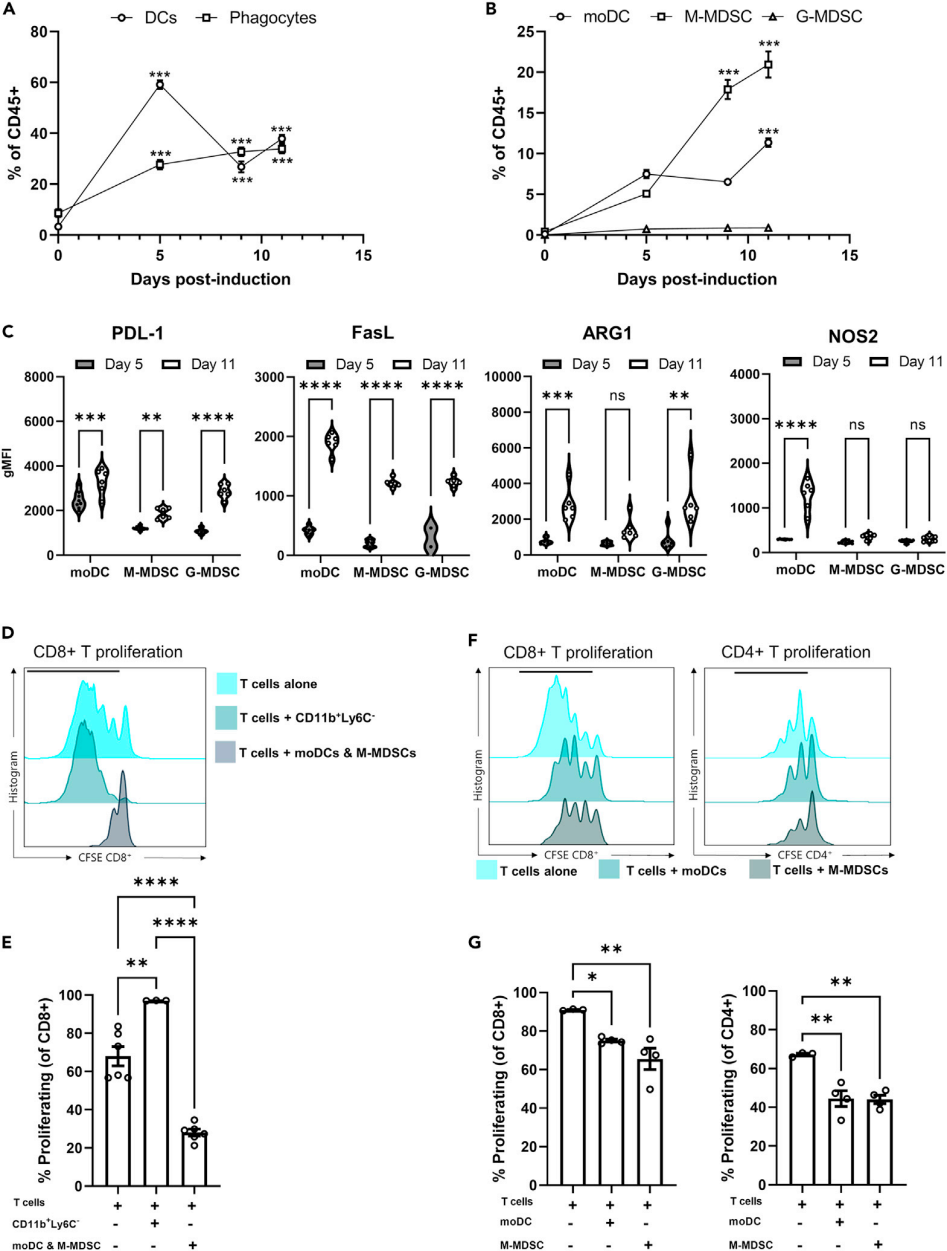
The myeloid compartment in B16.F10 melanoma shifts toward suppressive phenotypes as tumors develop (A) Flow cytometry quantification of the percentage of DCs (CD11c+) and phagocytes (CD11b+CD11c-) within CD45^+^ cells. (B) Flow cytometry quantification of MDSCs populations (moDCs; CD11b^+^CD11c^+^Ly6C^+^, M-MDSCs; CD11b^+^CD11c^−^Ly6C^+^, and G-MDSCs; CD11b^+^CD11c^−^Ly6G^+^) within CD45^+^ cells. (C) Flow cytometry quantification of the expression levels of immune-modulatory markers PD-L1, FasL, ARG1, and NOS2 by moDCs, M-MDSCs, and G-MDSCs. (D) Representative CFSE plots for CD8 T cell proliferation after culture alone, co-culture with tumor-derived CD11b^+^Ly6C^−^ cells, or co-culture with a mix of tumor-derived moDCs and M-MDSCs. Black bar highlights the gated proliferated cells. (E) Flow cytometry quantification of the percentage of proliferating CD8 and CD4 cells cultured alone, co-cultured with tumor-derived CD11b^+^Ly6C^−^ cells, or a mix of moDCs and M-MDSCs. (F) Representative CFSE plots for CD4 and CD8 T cell proliferation after co-culture with pre-sorted, tumor-derived moDCs or M-MDSCs. Black bar highlights the gated proliferated cells. (G) Quantification of T cell suppression following incubation with pre-sorted, tumor-derived moDCs and M-MDSCs compared to T cells cultured alone. Data are mean ± SEM; ∗∗ = p < 0.01, ∗∗∗ = p < 0.001, ∗∗∗∗ = p < 0.0001. (A–C) Mixed effect analysis with a Tukey’s *post hoc* test. (E and G) One-way ANOVA with a Tukey’s *post hoc* test. (A and B) n = 5 mice for day-5 and day-9 tumors and n = 6 for day-11 tumors, from two independent experiments comparing each cell type at day 5, 9, or 11 time points with the day 0 time point. (C) n = 8 mice for day 5 tumors and n = 6 mice for day 11 tumors from two independent experiments. (E) n = 3 and (G) n = 2 mice performed in duplicate from two different experiments.

The presence of distinct myeloid populations in melanoma has been well characterized (extensively reviewed by Veglia et al. and Barry et al.^25^^,^^56^). However, the kinetics of the infiltration and functions of each population as the tumor evolves are less well established. To address this in murine melanoma, we first characterized the myeloid compartment in healthy skin and compared it with the myeloid constituents present in small, palpable skin lesions at 5 days post-tumor induction and at day 9 and 11 post-induction, when tumors were more established (Figure S1A). In healthy skin, DCs (CD11c^+^) and phagocytes (CD11b^+^CD11c^−^) comprised a small proportion of the immune infiltrate. However, early B16.F10 lesions (day 5) exhibited a significant increase in the proportion of both populations, comprising ∼80% of the total immune infiltrate compared to healthy skin at day 0 (Figures 1A and S1B). The increased phagocyte infiltration was maintained at day 9 and 11 post-induction compared to normal skin (Figure 1A). In contrast, the abundance of DCs decreased sharply as tumors progressed, to a level comparable with phagocytes (Figures 1A and S1B). The shift in the dominance of specific myeloid populations may reflect differences in the function of myeloid components as tumors evolve. A more in-depth analysis of specific myeloid populations revealed that moDCs (CD11b^+^CD11c^+^Ly6C^+^) and M-MDSCs (CD11b^+^CD11c^−^Ly6C^+^) both significantly expanded throughout tumor progression compared to healthy skin at day 0 (Figure 1B). However, the M-MDSCs expanded more dramatically than moDCs to become the dominant myeloid population in established day-11 tumors. Surprisingly, the proportion of G-MDSCs (CD11b^+^CD11c^−^Ly6G^+^) slightly increased as tumors developed, but they represented a minor component of the infiltrate compared to M-MDSCs and moDCs (Figure 1B).

We then examined the wider myeloid compartment, observing a decrease in the percentage of cDC1 and cDC2 cells (Figure S1B) from day 5 to day 11 post-tumor induction. Within the defined moDC and M-MDSC populations, CX3CR1, marking monocytes and monocyte precursors, and the maturation marker F4/80, used to distinguish macrophages from DCs, were assessed (Figures S1C and S1D). Interestingly, approximately 95% of moDC were F4/80^+^ in day-5 tumors, and this proportion significantly decreased by day 11 (Figure S1C). Similarly, M-MDSCs also showed a decrease in the proportion of F4/80^+^ cells in day-11 tumors albeit to a lesser extent than the moDCs. CX3CR1 is expressed by most myeloid constituents and was highly expressed by both moDC and M-MDSCs. Surprisingly, the proportion of CX3CR1^+^ moDCs slightly increased in day-11 tumors while in M-MDSCs the proportion significantly decreased. These changes in F4/80 and CX3CR1 expression from day-5 to day-11 tumors likely reflect a shift in the phenotype of the moDCs and M-MDSCs to a more immature-like state.

As the recruitment of M-MDSCs and moDCs significantly increased in the evolving tumor, we then sought to understand how they contribute to the changing immune landscape and suppressive microenvironment. By immune phenotyping, we observed an upregulation in the expression of T cell-suppressive molecules, PD-L1, Fas Ligand (FasL), and ARG1 across moDC, M-MDSC, and G-MDSC subsets from day 5 to day 11 post-tumor induction (Figure 1C). Notably, moDCs were the only population to significantly upregulate NOS2 levels as tumors progressed (Figure 1C), suggesting that, while the expression of certain suppressive molecules is shared among myeloid populations, cell-type-specific phenotypes also exist which may critically influence their function. These data indicate that myeloid populations, particularly moDCs and M-MDSCs, which expand as tumors progress, upregulate T cell-suppressive molecules that may contribute to the onset or progression of an immunosuppressive microenvironment within the tumor.

To determine whether acquisition of a suppressive phenotype by these populations translated to a functional inhibition of T cells, we isolated myeloid cells (CD11b^+^Ly6C^−^, moDCs, and M-MDSCs) from day-11 tumors and co-cultured them with activated CD8 T cells, measuring their proliferative capacity based on Carboxyfluorescein succinimidyl ester (CFSE) levels (Figure 1D). Co-culture with CD11b^+^Ly6C^−^ cells, which include cDCs and neutrophils, promoted the proliferation of CD8 T cells compared to T cells alone (Figure 1E) which is likely due to their expression of T cell co-stimulatory molecules and their antigen presentation capabilities. In contrast, co-culture with a mixture of moDCs and M-MDSCs significantly suppressed CD8 T cell proliferation compared to CD8 T cells cultured alone or those cultured with the remaining myeloid fraction (Figures 1D and 1E). Importantly, proliferation of CD8 T cells exposed to M-MDSCs was more potently suppressed than proliferation of those co-cultured with moDCs (Figures 1F and 1G). Interestingly, both populations also impaired CD4 T cell proliferation to comparable levels (Figures 1F and 1G). These data highlight that MDSCs and moDCs which express immunosuppressive markers can functionally suppress both CD4 and CD8 T cell proliferation within the tumor. Of note, the other myeloid components functioned to support T cell proliferation, thus, highlighting that the fine balance of myeloid cells present can determine whether a microenvironment is immunosuppressive or stimulatory. These data support the idea that the tumor promotes a shift in recruitment and phenotype of myeloid populations as tumors develop, toward a more suppressive milieu capable of impairing T cell proliferation.

### Tumor conditioning induces myeloid cells to develop a suppressive phenotype

**Figure 2:**
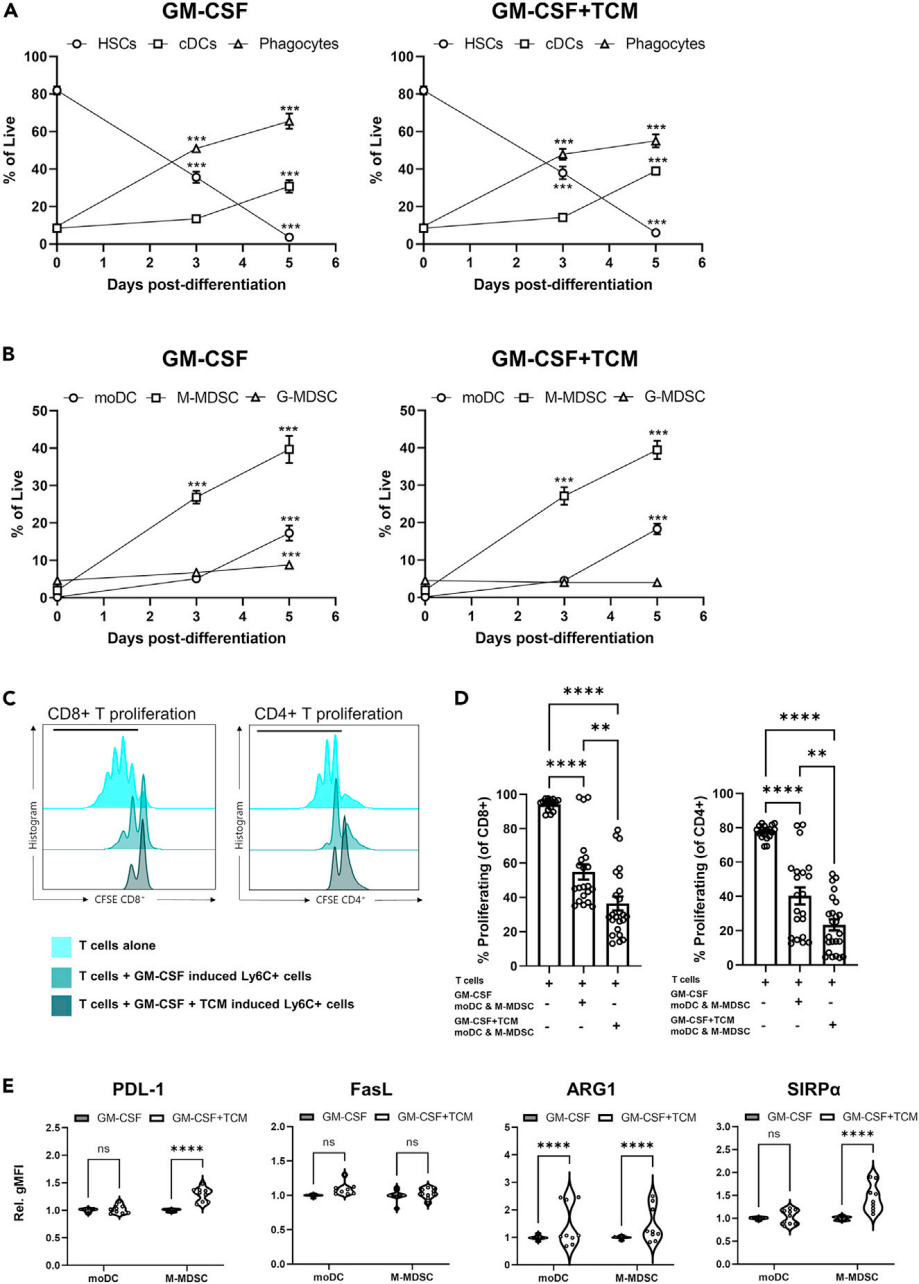
Tumor conditioning of myeloid cells *in vitro* recapitulates the myeloid compartment shift toward suppressive phenotypes Flow cytometry quantification of the differentiation of isolated SCA-1^+^ HSCs toward (A) cDCs and phagocytes and more specifically (B) moDCs, M-MDSC and G-MDSCs after treatment with either GM-CSF or GM-CSF supplemented TCM. (C) Representative CFSE plots for CD4 and CD8 T cell proliferation after incubation with *in vitro*-generated Ly6C^+^ myeloid cells in GM-CSF or GM-CSF-supplemented TCM compared to T cells cultured alone. Black bar highlights the gated proliferated cells. (D) Quantification of the percentage of proliferating CD8 and CD4 cells cultured alone, co-cultured with GM-CSF or GM-CSF supplemented TCM differentiated mixed moDCs and M-MDSCs. (E) Flow cytometry quantification of expression of immune-modulatory markers PD-L1, FasL, ARG1, and SIRP⍺ by moDCs and M-MDSCs. Data are mean ± SEM; ∗∗ = p < 0.01, ∗∗∗ = p < 0.001, ∗∗∗∗ = p < 0.0001. (A and B) Mixed effect analysis with a Tukey’s *post hoc* test. (D) One-way ANOVA with a Tukey’s *post hoc* test comparing each cell type at day 3 and 5 time points with the day 0 time point. (E) Two-way ANOVA with a Tukey’s multiple comparisons *post hoc* test. (A–C) n = 2 replicates for each condition from eight independent experiments. (D) n = 7 and (E) n = 3 independent experiments performed in triplicate.

To model development of suppressive myeloid populations within the tumor and assess whether tumor-derived factors could drive differentiation of myeloid cells toward an immunosuppressive phenotype, we utilized an *in vitro* culture system to generate different myeloid populations from hematopoietic stem cells (HSCs). Briefly, Sca-1^+^ bone marrow-derived HSCs were cultured in the presence of Granulocyte-macrophage colony-stimulating factor (GM-CSF) to maintain their growth, or GM-CSF supplemented with tumor cell-conditioned media (GM-CSF+TCM). At day 0, the culture was predominately composed of HSCs (Sca-1^+^CD11b^−^CD11c^−^, Figure S1E) and contained very small proportions of differentiated myeloid cells (Figures 2A and 2B). After 3 days of culture in the presence or absence of tumor-derived factors, the proportion of phagocytes significantly increased compared to normal day-0 cells (Figures 2A and S1F) with M-MDSCs comprising the dominant proportion of these cells (Figure 2B). The remaining cells comprised predominantly granulocytes, other DC populations, and a small proportion of lymphocytes. By day 5 post-differentiation, the cDC fraction had also expanded in both treatment conditions (Figures 2A and S1F) and comprised primarily moDCs, which also significantly increased compared to normal day-0 cells (Figure 2B). The phagocyte fraction continued to expand up to 5 days of differentiation, largely due to the expansion of M-MDSCs, while the proportion of G-MDSCs remained constant at all time points (Figures 2A and 2B). At this point in the differentiation process, very few HSCs remained (Figure 2A). These data strongly suggest that differentiation of HSCs toward myeloid constituents’ favors development of M-MDSCs and moDCs and an absence of G-MDSCs. Furthermore, treatment with tumor-derived factors does not directly influence the differentiation into these cell types in this *in vitro* culture system.

While myeloid composition was unaltered by TCM conditioning *in vitro*, functional alterations were apparent, with moDCs and M-MDSCs derived from TCM-treated HSC cultures capable of suppressing CD4 and CD8 T cell proliferation to a greater extent than those treated with GM-CSF alone (Figures 2C and 2D). In a similar manner to the *in vivo* tumor setting, TCM-treated M-MDSCs, which were the dominant cell type in these cultures, showed increased expression of PD-L1, ARG1, and SIRPα (Figures 2E and S1G). moDCs showed a slight trend toward increased PD-L1 and SIRPα expression and a significant increase in ARG1 expression following TCM treatment (Figures 2E and S1G). In contrast to *in vivo* tumor-derived moDCs and M-MDSCs, FasL was not upregulated upon TCM treatment, suggesting that, while the *in vitro* culture system mimics the *in vivo* development of suppressive myeloid populations, the mechanisms that mediate T cell suppression vary between the systems. Furthermore, these data show that tumor-derived factors can enhance the suppressive capability of myeloid constituents without directly influencing their differentiation and development.

### The CD47-SIRPα signaling axis drives myeloid cells toward an immunosuppressive phenotype

We next sought to determine the factors driving the suppressive phenotypes observed in M-MDSCs and moDCs. Previous reports have shown that CD47 binds to SIRPα, an immune checkpoint expressed mainly by myeloid cells. Engagement of these proteins prevents phagocytosis to limit the clearance of old cells or non-self-antigen-presenting cells. In tumors, reports have suggested that, once this pathway is triggered, impaired phagocytosis by antigen-presenting cells is accompanied by inhibition of their inflammatory activities and impaired clearance of dead or dying cells.^35^^,^^57^ These previous studies led us to consider whether loss of phagocytosis by engagement of this axis limits the pro-inflammatory, anti-tumorigenic phenotype of moDCs and M-MDSCs to favor suppression of tumor-infiltrating T cells.

**Figure 3:**
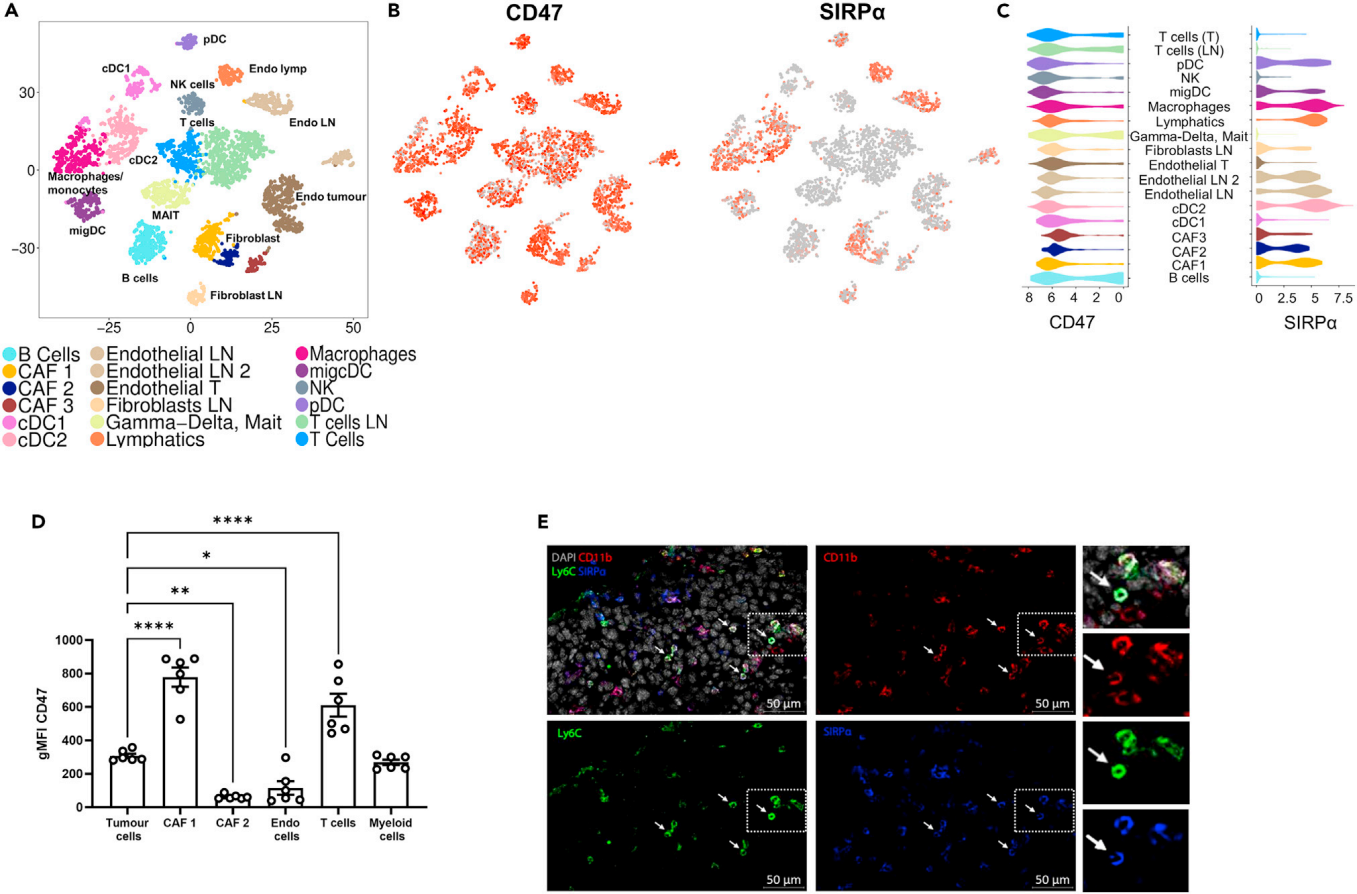
Distribution of CD47 and SIRPα expression across the TME (A) Clustering of stromal populations identified in B16.F10 melanomas and matched draining lymph nodes analyzed from data previously published by Davidson et al.^58^ (B) Expression of CD47 and its cognate receptor, SIRPα, distributed across stromal clusters. (C) Violin plots highlighting widespread CD47 but restricted SIRPα expression across stromal subsets. (D) Flow cytometry quantification of CD47 expression at the protein level in T cells, (immunomodulatory) CAF 1, (myofibroblast) CAF 2, myeloid cells, endothelial cells (CD31^+^), and B16.F10 tumor cells. (E) Representative confocal image of a day 11 B16.F10 melanoma showing myeloid populations. Arrows indicate CD11b+Ly6C + SIRPα+ cells. Insets show zoom of selected ROI. Arrowheads depict cells positive for CD11b but negative for Ly6C and SIRPα. DAPI (gray), CD11b (red), Ly6C (green), SIRPα (blue). Scale bar is 50μm. Data are mean ± SEM; ∗ = p < 0.05, ∗∗ = p < 0.01, ∗∗∗ = p < 0.001, ∗∗∗∗ = p < 0.0001. (D) One-way ANOVA with a Dunnett *post hoc* test. (D) n = 3 replicates from two independent experiments.

To test this, we first examined a published single-cell RNA sequencing (scRNA-seq) dataset^58^ characterizing the non-cancer-cell components of the B16.F10 TME (Figure 3A) to determine the distribution of CD47 and SIRPα within the tumor stroma. CD47 was diffusely expressed across both immune and non-immune stromal constituents at the RNA level (Figures 3B and 3C). In contrast, SIRPα expression was more exclusive, restricted to the myeloid, endothelial, and immune-modulatory cancer-associated fibroblast (CAF) compartments of the tumor (Figures 3B and 3C). CD47 distribution within the TME was confirmed at the protein level in dissociated tumors (Figure 3D). Similarly, CD47 protein was widely detected across multiple cell compartments, including tumor cells, CAFs, endothelial cells, and myeloid and T cells. However, interestingly, the dominant signal within the microenvironment came from T cells^57^^,^^59^ and immunomodulatory CAFs (CAF1; Figure 3D). SIRPα-expressing cells were predominantly from the myeloid lineage, and these SIRPα^+^ cells were abundant throughout the tumor core (Figure 3E). This suggests that engagement of myeloid cell SIRPα would be predominantly driven via interactions with other stromal components expressing CD47 in addition to those with tumor cells. These data indicate that CD47-SIRPα signaling could be a mechanism contributing to the suppressive effects of myeloid cells within established tumors.

**Figure 4:**
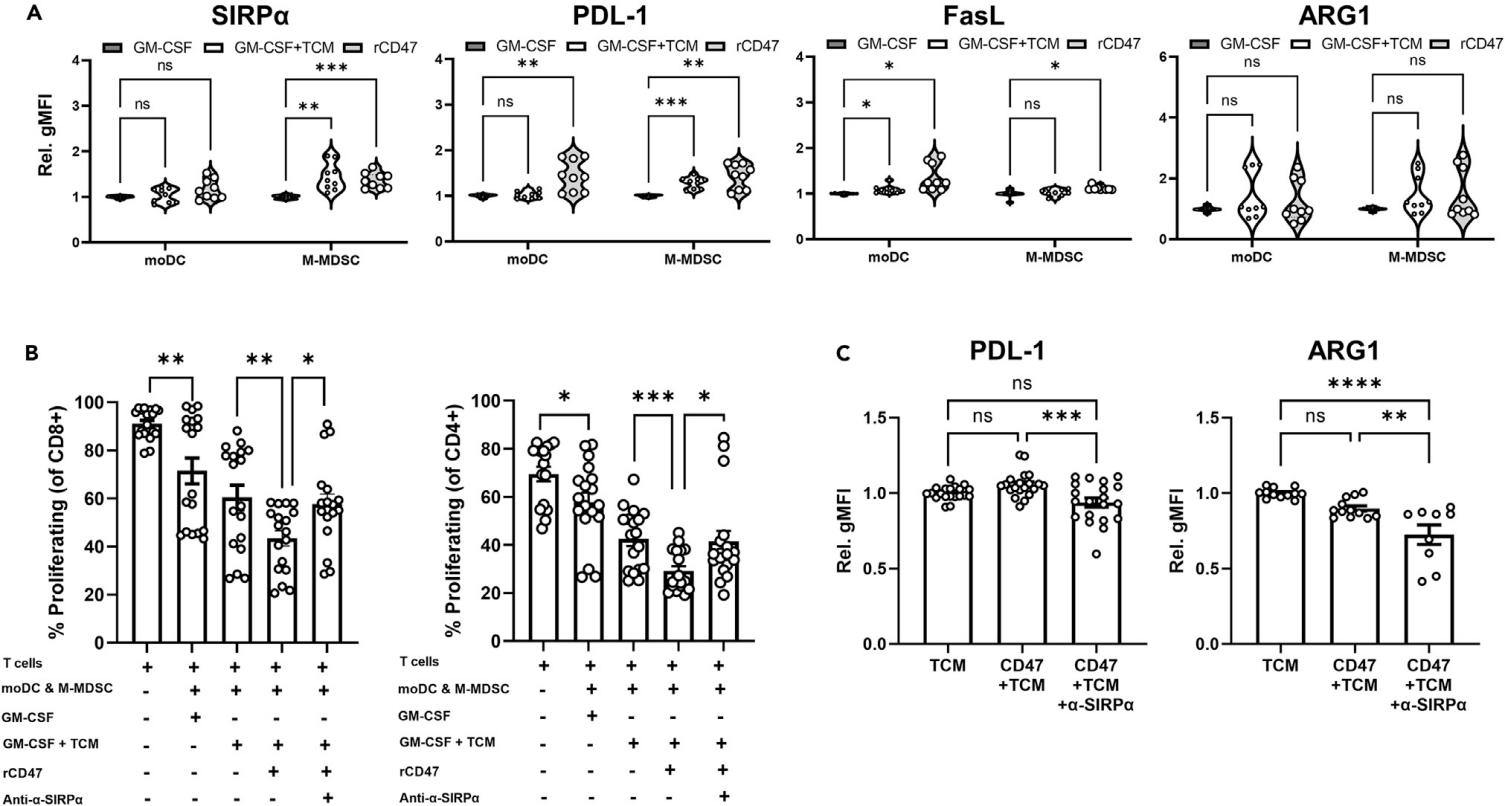
CD47 stimulation promotes a suppressive phenotype in myeloid populations and is rescued by SIRPα blockade (A) Flow cytometry quantification of expression levels of immune modulatory markers PD-L1, FasL, ARG1, and SIRP⍺ by moDCs and M-MDSCs following stimulation with TCM or rCD47 (expressed as relative gMFI). (B) Flow cytometry quantification of the percentage of proliferating CD8 and CD4 cells measured by CFSE; cultured alone or co-cultured with a mixture of moDCs and M-MDSCs pre-treated with different combinations of TCM, rCD47, and anti-SIRP⍺. (C) Flow cytometry quantification of expression of PD-L1 and ARG1 by mixed moDC and M-MDSC cultures after treatment with TCM with or without rCD47 and anti-SIRP⍺. Data are mean ± SEM; ∗ = p < 0.05, ∗∗ = p < 0.01, ∗∗∗ = p < 0.001, ∗∗∗∗ = p < 0.0001. (A) Two-way ANOVA with a Tukey’s multiple comparisons *post hoc* test. (B) Unpaired t test. (C) One-way ANOVA with a Tukey’s *post hoc* test. (A) three independent experiments each with n = 3 replicates. (B) n = 6 independent assays performed in triplicate. (C) n = 6 independent assays for PD-L1 and n = 3–4 independent assays for ARG1, each performed in triplicate.

To then determine if CD47-SIRPα signaling plays a role in the acquisition of a suppressive phenotype and mediating immunosuppression, we measured the influence of CD47 stimulation on expression of T cell checkpoint molecules by moDCs and M-MDSCs *in vitro*. Stimulation with active recombinant CD47 peptide (rCD47) augmented the suppressive phenotype of moDC and M-MDSCs compared to GM-CSF and slightly enhanced the effects of TCM treatment, with elevated expression of PD-L1, FasL, V-domain immunoglobulin suppressor of T cell activation (VISTA), and Indolamine-2,3-Dioxygenase (IDO) (Figures 4A, S2A, and S2B). We also observed a significant increase in SIRPα expression by M-MDSC upon CD47 treatment (Figure 4A), indicating that CD47 ligation may operate in a feedback loop to boost SIRPα availability, further sensitizing myeloid cells to CD47 signals and suppressive functions such as inhibition of phagocytosis.

The induction of a more suppressive phenotype through CD47 stimulation was accompanied by an enhanced capacity to inhibit CD8 and CD4 T cell proliferation (Figure 4B). Again, while TCM-treated myeloid populations were more potent than GM-CSF at inhibiting T cell proliferation, this was further augmented following CD47 stimulation and engagement of SIRPα on the MDSCs (Figure 4B). When CD47-SIRPα interactions were disrupted with a SIRPα-blocking antibody, any enhanced suppression driven by CD47 engagement was effectively lost (Figure 4B). However, suppression was not fully reversed by α-SIRPα blockade implying that SIRPα sites were not fully occupied, or that other factors also contribute to the suppressive potential of tumor-conditioned myeloid cells. Indeed, a concurrent reduction in PD-L1 and ARG1 expression was detected in mixed moDC and M-MDSC cultures when SIRPα was blocked in the presence of rCD47 compared to CD47-stimulated MDSCs alone (Figure 4C).

### Blockade of the CD47-SIRPα interaction restores the phagocytic capabilities of myeloid cells

**Figure 5:**
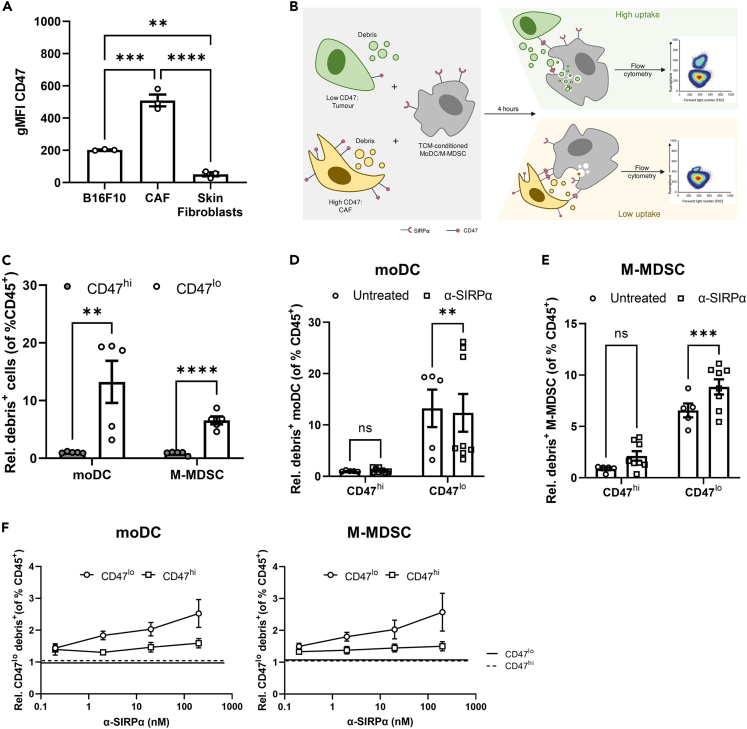
Blockade of CD47-SIRPα interaction boosts the phagocytic capabilities of MDSCs (A) Flow cytometry quantification of CD47 expression levels by B16.F10 melanoma, CAFs, and dermal fibroblasts. (B) Schematic representation of the assay developed to measure the effects of CD47 on the phagocytic potential of different myeloid subsets. M-MDSCs and moDCs were mixed with a mix of fluorescently labeled CD47-high or -low expressing cells and their cell debris. Uptake of fluorescent debris by moDCs and M-MDSCs was then analyzed by flow cytometry and quantified as the proportion of CD45^+^ cells that are moDCs or M-MDSCs that have phagocytosed cell debris. (C) Flow cytometry quantification of uptake of labeled CD47-high or -low cell debris by moDCs and M-MDSCs. Quantification of phagocytosis by (D) moDCs and (E) M-MDSCs after co-culture with CD47^lo^ or CD47^hi^ cells in the presence or absence of SIRPα neutralizing antibody. (F) Competition assay showing occupation of SIRPα epitopes by anti-SIRPα (P84) antibody. Upper panel: quantification of gMFI signal detected for fluorophore conjugated anti-SIRPα antibody after epitope blockade by Ultra-LEAF- SIRPα antibody. Lower panel: quantification of gMFI signal detected for Ultra-LEAF antibody detected by fluorophore conjugated Rat IgG. Data are mean ± SEM; ∗∗ = p < 0.01, ∗∗∗ = p < 0.001, ∗∗∗∗ = p < 0.0001. (A, C, D, E) One-way ANOVA with a Tukey’s *post hoc* test. (A) n = 1 performed in triplicate. (C–E) n = 3 independent experiments performed in duplicate, (F) n = 3 performed in triplicate.

Having observed that perturbation of CD47-SIRPα signaling reduced the levels of T cell-suppressive molecules and partially restored T cell proliferative potential, we then examined whether blockade of SIRPα signaling also enhanced the phagocytic functions of the myeloid cells. To do this, we modified our *in vitro* system. First, moDCs and M-MDSCs were differentiated in GM-CSF+TCM for 5 days to mimic tumor conditioning. We then incorporated cells found within a tumor which either express high (CAFs) or low (B16.F10 tumor cells) levels of CD47 as determined by flow cytometry (Figures 5A and S3A). The cells were fluorescently labeled, and 25% were killed by heat treatment to generate a mix of live cells and labeled debris. The capacity of moDCs and M-MDSCs to uptake debris in the presence or absence of CD47 was then quantified by flow cytometry (Figures 5B and S3B). As expected, both moDC and M-MDSCs displayed enhanced phagocytosis (as measured by fluorescent signal detected within myeloid cells) when in the presence of CD47^low^ tumor cells compared with CD47^high^ CAFs (Figure 5C). Prior to testing whether SIRPα blockade could enhance the phagocytic capacity of moDCs and M-MDSCs, we confirmed that the SIRPα-blocking antibody could efficiently bind and occupy all available epitopes. To do this, we performed an antibody competition assay on Ly6C^+^ cells (Figure S3C). Briefly, after treatment with the anti-SIRPα-blocking antibody, the SIRPα epitope was no longer detectable by conjugated antibodies, indicating that prior treatment with the blocking antibody effectively covered all epitopes and thereby limited detection with the conjugated antibody (Figure S4B, left panel). Application of a conjugated anti-rat immunoglobulin G (IgG) effectively recognized the backbone of the blocking antibody, confirming that it had reached its target, was occupying the SIRPα site, and had not been internalized (Figure S3C, right panel).

Perturbation of the anti-phagocytic signal induced by SIRPα blockade had little impact on uptake of cellular material by moDCs in the presence of either CD47^high^ or CD47^low^ cells with low doses of SIRPα-blocking antibody (Figure 5D). In contrast, even at the lowest concentrations tested, pre-treatment of M-MDSCs with anti-SIRPα significantly boosted their phagocytic capacity (Figure 5E). This may have been a consequence of sub-maximal occupancy by the blocking antibody. Indeed, when titrated, a dose-dependent enhancement of phagocytosis on M-MDSCs and moDCs could be detected with increasing concentrations of blocking antibody (Figure 5F).

These data highlight that myeloid cells recruited to the tumor enter at the periphery where they likely encounter an environment rich in CD47-expressing cells (such as CAFs). This engages SIRPα on the myeloid cells, skewing them toward a suppressive phenotype and inhibiting their potential to phagocytose tumor cell-derived material. As a result, this could limit their capacity to present antigen and/or express critical anti-tumorigenic cytokines. The cells in turn acquire a suppressive phenotype that impairs T cell reactivity to the tumor. Blocking this interaction on M-MDSCs and moDCs may increase tumor cell clearance and reduce expression of T cell-suppressive molecules.

### CD47-SIRPα signaling induces changes in cellular energetics

**Figure 6:**
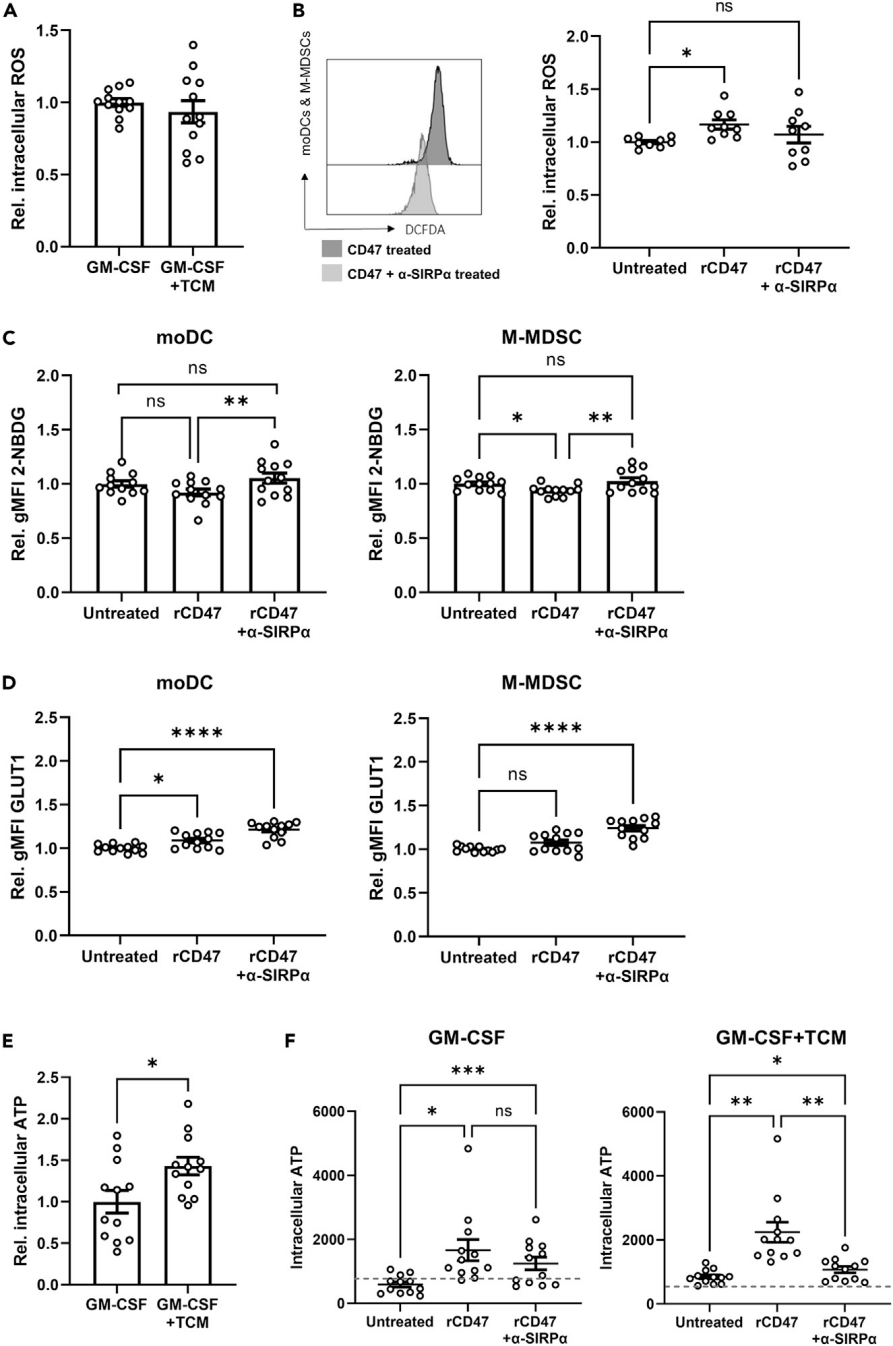
CD47-SIRPα modulation enhances phagocytosis and modifies cellular energetics (A) Flow cytometry quantification of intracellular DCFDA (ROS) signal detected in mixed moDCs and M-MDSCs grown in GM-CSF or GM-CSF-supplemented TCM. (B) Representative flow cytometry histograms showing DCFDA signal in moDCs and M-MDSCs in the presence of CD47 with (dark gray) or without (light gray) anti-SIRPα and quantification of DCFDA gMFI signal in each condition. (C) Quantification of uptake of the glucose analogue 2-NBDG *in vitro* by differentiated moDCs and M-MDSCs with or without CD47 stimulation in the presence or absence of anti-SIRPα. (D) Flow cytometry quantification of the glucose transporter GLUT1 expression on the surface of *in vitro*-differentiated moDCs and M-MDSCs with or without CD47 stimulation in the presence or absence of anti-SIRPα. (E) Quantification of intracellular ATP levels in mixed moDC and M-MDSC cultures grown in GM-CSF or GM-CSF-supplemented TCM. (F) Quantification of intracellular ATP driven levels in mixed moDC and M-MDSC cultures grown in GM-CSF or GM-CSF-supplemented TCM with or without CD47 stimulation in the presence or absence of anti-SIRPα. Data are mean ± SEM; ∗ = p < 0.05, ∗∗ = p < 0.01, ∗∗∗ = p < 0.001, ∗∗∗∗ = p < 0.0001. All data were normalized to the GM-CSF or untreated sample. (A, E) Paired t test. (B-D) One-way ANOVA with Dunnett’s *post hoc* test. (F) One-way ANOVA with a Tukey’s *post hoc* test. (A) Assays n = 4 independent experiments each performed in triplicate. (B) n = 3 independent experiments each performed in duplicate. (C and D) n = 4 independent experiments each performed in triplicate. (E) n = 4 independent experiments each performed in triplicate. (F) n = 3 independent experiments each performed in triplicate.

We subsequently examined whether SIRPα-mediated inhibition of phagocytosis in myeloid cells induces a suppressive phenotype through altering the metabolic state of the MDSCs. ROS production by tumor-infiltrating immune cells has been correlated with more immunosuppressive phenotypes.^28^^,^^60^^,^^61^ *In vitro* differentiated moDC and M-MDSC expressed intracellular ROS to equivalent levels in the presence or absence of tumor-derived factors (Figure 6A). Interestingly, rCD47 stimulation induced a small but significant increase in ROS generation by TCM-conditioned moDCs and M-MDSCs, and SIRPα blockade restored the level to baseline (Figure 6B). These data show that enhanced immunosuppression induced by CD47-SIRPα interaction is associated with an increased intracellular ROS. Importantly, TCM conditioning alone did not enhance ROS production and additional rCD47 stimulation was required. This led us to speculate that, while tumor-derived factors are critical for regulating the immunosuppressive phenotype of moDCs and M-MDSCs, engagement with CD47 expressed by the TME may be needed to enhance a metabolic shift in these cells.

Considering the shift in ROS production alongside recent reports indicating that glycolysis might provide the energetic intermediates required for immune activation and antigen presentation,^62^^,^^63^ we then examined the effects of CD47-SIRPα on glycolysis. Indeed, phagocytosis mediated by engagement of CD47-SIRPα was accompanied by a small but significant decrease in glucose uptake (measured by 2-( N- (7-nitrobenz-2-oxa-1,3-diazol-4-yl)amino)-2-deoxyglucose (2-NBDG) uptake) for both moDC and M-MDSCs (Figure 6C) and was restored with SIRPα blockade (Figure 6C). Interestingly, rCD47 stimulation of moDCs and M-MDSCs induced an increase in surface expression of GLUT1, the main transporter responsible for glucose uptake, and this was further enhanced in the presence of SIRPα blockade (Figure 6D).

Further evidence suggests that tumor-infiltrating myeloid populations can modulate their activation state by increasing the synthesis and secretion of ATP where it is rapidly catabolized into adenosine.^64^^,^^65^ Its accumulation in solid tumors then impairs anti-tumor T cell responses.^66^ We therefore looked further downstream to a general metabolic energetic marker, measuring the total ATP production as an indicator of the energetic state of cells. The more suppressive TCM-treated moDCs and M-MDSCs displayed a greater accumulation of ATP compared with GM-CSF-treated cells in line with reports of impaired immunity (Figures 6E and 6F). Additionally, stimulation with CD47 induced a further, significant accumulation of intracellular ATP which was effectively abrogated by anti-SIRPα treatment in both GM-CSF- and TCM-treated conditions (Figure 6F).

Together, these data indicate that CD47-SIRPα engagement reduced the energetic requirements of moDCs and M-MDSCs, and this was associated with acquisition of a more suppressive phenotype. This shift in metabolic state was reversed upon SIRPα blockade.

### Anti-SIRPα therapy restores antigen uptake, processing, and presentation in tumors

**Figure 7:**
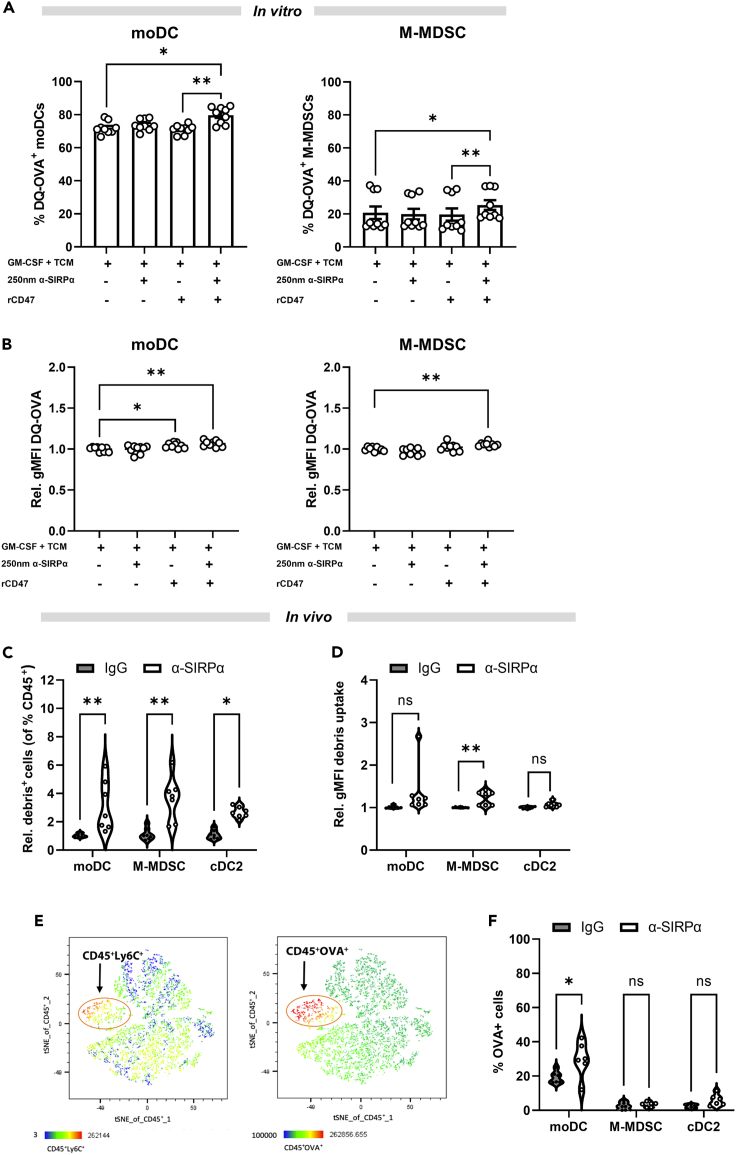
SIRPα therapy induces phagocytosis, antigen processing, and presentation in myeloid cells (A) Flow cytometry quantification of the frequency of GM-CSF-supplemented-TCM-treated moDCs and M-MDSCs that uptake and proteolytically process DQ-OVA antigen with or without CD47 stimulation, in the presence or absence of anti-SIRPα. (B) Quantification of the levels of DQ-OVA processed by moDCs and M-MDSCs (gMFI). Data normalized to GM-CSF-TCM condition. (C) Quantification of the abundance of moDCs, M-MDSCs and cDC2s that phagocytosed GFP^+^ melanoma-derived material *in vivo* following therapeutic blockade of CD47-SIRPα signaling. Expressed as the proportion of CD45^+^ cells that are moDCs or M-MDSCs that have phagocytosed GFP+ cell debris. (D) Quantification of the level of GFP material ingested by moDCs, M-MDSCs, and cDC2s. For (C) and (D), data were normalized by the signal detected in the rat IgG1 injected conditions. (E) Representative tSNE plots derived from the flow cytometry data (of total CD45^+^ cells) showing that intratumoral CD45^+^Ly6C^+^ cells exhibit the highest level of SIINFEKL (OVA antigen) complexed with MHCI. (F) Quantification of presentation of the OVA peptide SIINFEKL by moDCs, M-MDSCs, and cDC2s that was acquired by uptake and processing of material from B16.OVA melanoma cells. Data are mean ± SEM; ∗ = p < 0.05, ∗∗ = p < 0.01. (A and B) One-way ANOVA with Tukey’s multiple comparisons and Dunnett’s *post hoc* test, respectively. (C, D, and F) Two-way ANOVA with Tukey’s multiple comparisons test. (A and B) n = 3 performed in triplicate; (D and F) two independent experiments of n = 4 mice each.

We next sought to determine whether disrupting the CD47-SIRPα interaction in moDCs and M-MDSCs and the accompanying changes in cell phenotype translated to an activation of pro-inflammatory functions, restoration of phagocytosis, and antigen uptake, processing, and presentation. Using the *in vitro* culture system, we identified that, in addition to enhanced uptake of cellular debris after SIRPα blockade (Figure 5), antigen processing was also enhanced. All myeloid subsets tested could proteolytically cleave the modified version of Ovalbumin (OVA), DQ-OVA, with cDC2s and moDCs being more efficient than M-MDSCs (Figures 7A, S4A, and S4B). However, the proportion of moDCs, M-MDSCs, and cDC2s processing DQ-OVA significantly increased after SIRPα blockade, only in the presence of rCD47 (Figures 7A, S4A, and S4B). The levels of processing within moDCs and M-MDSCs, and to a lesser extent cDC2s, also increased (Figures 7B and S4C). This shows that antigen processing, both in terms of the number of cells processing and the amount of antigen processed by individual myeloid cells after rCD47 ligation, was enhanced by anti-SIRPα treatment.

Having shown the effects of SIRPα blockade on the antigen sampling and processing capacity *in vitro*, we then examined the effects of SIRPα blockade on these functions within tumors *in vivo*. Following anti-SIRPα treatment of B16.F10.GFP tumor-bearing mice (melanoma cells overexpressing GFP; Figure S4D), we detected a significant increase in the frequency of moDCs, M-MDSCs, and cDC2s sampling tumor-derived material as determined by detection of tumor-derived GFP signal within the cells (Figures 7C and S4E). The amount of tumor-cell debris engulfed by myeloid cells also increased upon SIRPα blockade (Figure 7D), actively showing that SIRPα blockade enhances both the number of tumor-associated myeloid cells sampling material and the phagocytic capacity of individual cells *in vivo*. Lastly, in B16.F10 tumors which overexpressed cytoplasmic OVA, SIRPα blockade resulted in increased presentation of the OVA antigenic peptide SIINFEKL complexed with Major histocompatibility complex I (MHCI) on the surface of moDCs, M-MDSCs, and cDC2s (Figures 7E and 7F). These data suggest that blocking SIRPα restores the ability of moDCs and M-MDSCs to phagocytose dead or dying tumor cells *in vivo* and then proteolytically process and present tumor antigen to infiltrating T cells.

### Blockade of SIRPα signaling alters the immune landscape and slows the growth of established tumors

**Figure 8:**
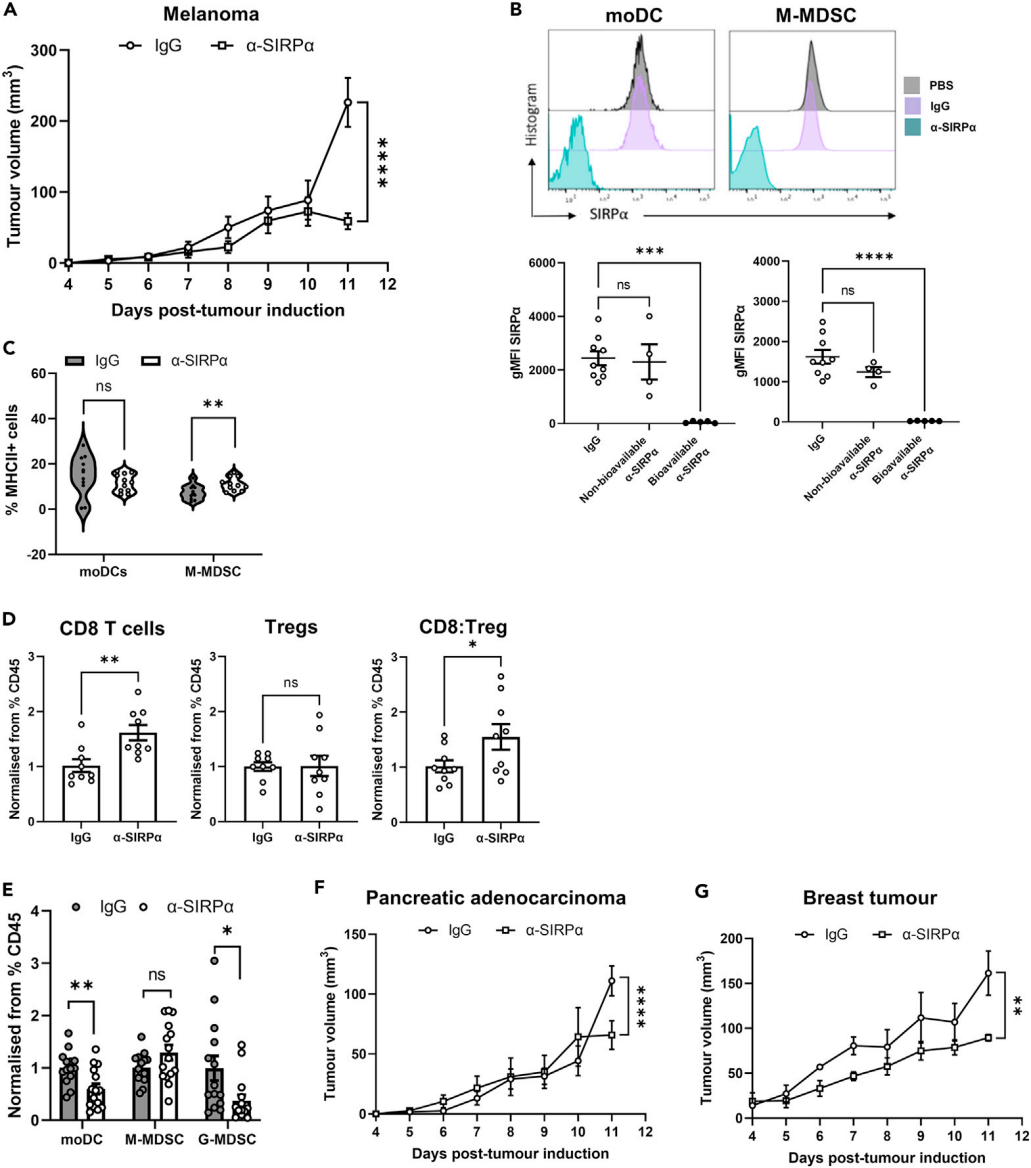
Therapeutic SIRPα blockade slows growth of established tumors *in vivo* (A) Volume (mm^3^) of B16.F10 melanomas grown in mice treated with rat-IgG1 (isotype control) or anti-SIRPα. (B) Representative flow cytometry histograms and quantification of expression levels showing SIRPα bioavailability at the tumor site in untreated, isotype or anti-SIRPα treated mice. Only mice in which SIRPα signal was reduced were included in the analyses. (C) Flow cytometry quantification of MHCII expression by tumor-infiltrating moDCs and M-MDSCs after treatment with anti-SIRPα or isotype. (D) Flow cytometry quantification of CD8^+^ T cells and Tregs (normalized to the percentage of CD45^+^ cells) and the ratio of CD8:Tregs. (E) Quantification of tumor-infiltrating moDCs, M-MDSC, and G-MDSCs (normalized to the percentage of CD45^+^ cells) after treatment with anti-SIRPα or isotype control. Data were normalized to the rat-IgG1 controls. (F) Volume (mm^3^) of subcutaneously injected pancreatic ductal adenocarcinoma or orthotopic E0771 breast tumors treated with IgG or anti-SIRPα over 11 days. Data are mean ± SEM; ∗ = p < 0.05, ∗∗ = p < 0.01, ∗∗∗ = p < 0.001, ∗∗∗∗ = p < 0.0001. (A and D–G) Paired t test. (B and C) One-way ANOVA with a Dunnett’s *post hoc* test. (A–C and F and G) n = 14 (rat-IgG1) and n = 15 (anti-SIRPα-P84) from 5 independent experiments. (D) n = 3 for each group from 3 independent experiments.

Since SIRPα blockade reverts MDSCs to a less suppressive phenotype and promotes phagocytosis, antigen processing, and presentation of tumor-derived material (Figures 7C–7F), we then tested the impact of SIRPα blockade, and its affects, on B16.F10 tumor development *in vivo*. Mice bearing palpable tumors received anti-SIRPα antibody or IgG control at days 5 and 8 post-tumor induction (when moDCs and M-MDSCs dominate the myeloid infiltrate). Blockade of SIRPα significantly impaired the growth of established tumors by day 11 post-tumor induction compared with isotype control-treated mice (Figure 8A). Importantly, within each experiment we tested the bioavailability of SIRPα after treatment with the blocking antibody. Here, loss of detectable SIRPα signal confirmed that the blocking antibody treatment had penetrated the tumor tissue and was occupying SIRPα epitopes on myeloid cells (Figure 8B).

The suppression of tumor growth with SIRPα blockade was accompanied by a remodeling of the immune microenvironment. A significant increase in MHCII^+^ M-MDSCs was detected, suggesting that these cells had become more mature upon treatment (Figure 8C). This correlated with an increase in CD8 T cell abundance and an increased CD8:Treg ratio, typical of improved prognosis (Figure 8D).^67^ Increased cytotoxic T cell infiltration in the presence of myeloid cells with enhanced antigen presentation capability (Figures 7E and 7F) likely contributed to the impairment of tumor growth. Further examination of myeloid composition showed that there was a decrease in the proportion of moDCs and G-MDSCs in treated mice compared to control (Figure 8E). While no difference in M-MDSCs recruitment was observed (Figure 8E), we observed a slight increase in the relative proportion of M-MDSCs expressing CX3CR1 and a significant increase in CX3CR1 expression (Figures S5B and S5C). This coincided with an increase in cDC1s, thus shifting the tumor back toward a phenotype reminiscent of that observed in the early day-5 lesions (Figures 1A and S5A). These data suggest that, in addition to a restoration of myeloid cell phagocytic potential along with enhanced antigen processing and presentation capabilities, SIRPα blockade partially restores M-MDSC maturation and reduces the recruitment of suppressive myeloid cell subtypes into the tumor.

Lastly, to determine if this was a melanoma-specific myeloid response, we treated mice bearing syngeneic pancreatic^68^ or breast tumors^69^ (that express similar or higher levels of CD47 compared to B16.F10 cells, Figure S5D) using the same treatment regimen. As observed in melanoma, blockade of SIRPα supported a significant slowing of tumor growth in both tumor models (Figures 8F and 8G). Together these data indicate that disruption of the CD47-SIRPα signaling axis modulates myeloid composition and functionality toward a pro-inflammatory state. These cells are more capable of uptake, processing, and presentation of tumor-derived material to infiltrating T cells, which, in the absence of suppressive mediators, exerts cytotoxic activity against the tumor and supports tumor resolution.

## Discussion

While immunotherapies have changed the landscape of cancer therapy, many patients fail to mount a long-term response to current therapies and in many cases experience toxicities due to overt T cell activation.^9^^,^^70^ Therefore, targeting molecules that modulate phagocytosis and antigen presentation by myeloid cells alone or in combination with T cell immune checkpoint therapy may mitigate some of these toxicities. This dual targeting approach would likely support an effective anti-tumor immune response.

Here we have shown how SIRPα expressing myeloid cells encounter a CD47-rich microenvironment as they infiltrate the tumor. As T cells and CAFs, the main source of CD47 in the melanoma model, are predominantly located at the tumor edge along the boundary with healthy tissue, it is likely that engagement of CD47 by myeloid cells occurs as the cells first enter the tumor before they penetrate the core. Engagement of SIRPα with CD47 on myeloid cells^36^^,^^38^^,^^57^ contributes to the generation of an immune-suppressive environment by reducing sampling, processing, and presentation of tumor-derived antigen to infiltrating T cells. Furthermore, it augments expression of molecules involved in mediating T cell suppression, namely FasL, PD-L1, and IDO. Disruption of this pathway induced significant changes in recruitment and activity of multiple myeloid populations, tipping the balance from suppression toward inflammation, and was sufficient to slow tumor growth by increasing tumor cell phagocytosis and a subsequent increase in cytotoxic T cell infiltration.

To date, the majority of work to target phagocytosis has focused on disruption of CD47-SIRPα through the blockade of CD47, with numerous phase 1–2 clinical trials underway in hematological malignancies and solid tumors.^21^^,^^50^^,^^71^^,^^72^ However, this may prove problematic as many normal cells also express CD47, including red blood cells and platelets, and as such significant off-target toxicities have been reported. Thus SIRPα and its more restricted expression is an attractive alternative therapeutic target, with potentially lower toxicity.^40^^,^^44^^,^^73^ Indeed, we showed that, even in an aggressive melanoma tumor model, SIRPα blockade dampened the inhibitory signal which contributed to the myeloid cells suppressive state and induced higher T CD8^+^:T_reg_ ratio within the TME. Consistent with work from Matozaki and colleagues, who showed that treatment of renal tumors with a high-affinity anti-SIRPα antibody reduced tumor volume and increased CD8 T cell infiltration^74^ and macrophages,^75^ we showed a similar phenomenon (using a different antibody clone) in murine models of melanoma, pancreatic adenocarcinoma, and breast adenocarcinoma. This shows that modulation of myeloid cell phagocytosis is a key regulator of anti-tumor immunity that is dysregulated in multiple tumor types; however this has only been interrogated in a single mouse model per tumor type. Furthermore, CD47-SIRPα interactions increased the suppressive capacity of myeloid populations *in vitro*, but its disruption with a SIRPα-neutralizing antibody shifted the cells to a more pro-inflammatory state. There was a concomitant reduction in expression of key immunosuppressive molecules and a reversion of the inhibition of T cell proliferation induced by a CD47-rich TME.

Our data indicate that modulation of the suppressive myeloid state may at least be in part driven by changes to the phagocytosis pathway, and capacity to present antigen. Indeed, when myeloid cells encountered a CD47-rich environment, impaired phagocytosis of tumor cell debris was observed. Treatment with the SIRPα-neutralizing antibody boosted sampling of material, particularly by M-MDSCs, as well as enhanced antigen processing and cross-presentation on MHCI. Changes in functional state were further supported by an altered glucose uptake and redistribution of cellular ATP. Less phagocytosis and cellular processing seen with CD47-mediated acquisition of a more suppressive phenotype were reflected in reduced energetic requirements and ATP accumulation. Hammami et al. previously demonstrated that bone marrow-derived MDSCs increased ATP and Nicotinamide adenine dinucleotide phosphate (NADPH) production during maturation, indicating deterioration of metabolic activity, and development of an immunosuppressive state.^76^ Accordingly, we observed that CD47 was contributing, in part, to establishing an immunosuppressive state which was reversed upon anti-SIRPα treatment and reactivation of more pro-inflammatory function. These findings are consistent with recent work by Baumann et al. who reported that metabolism and energetic state correlated with MDSC-mediated T cell suppression through passage and accumulation of toxic metabolites within the cells.^77^ Consequently, rather than modulating accumulation of myeloid cells to impact tumor clearance, SIRPα blockade instead favors myeloid reprogramming to drive activation and tumoricidal function of other infiltrating populations, such as CD8 T cells and cDC1s.

This work contributes to the growing body of evidence showing that combining SIRPα blockade as an innate checkpoint inhibitor with anti-PD1 or CTLA4, which targets T cell exhaustion, may improve therapeutic efficacy by boosting antigen uptake and presentation to enhanced numbers of reawakened T cells.

### Limitations of the study

We show here that disruption of CD47-SIRPα axis through inhibition of SIRPα on myeloid cells supports a restoration of phagocytosis, antigen processing and presentation capacity, and a more mature and activated immune phenotype and is accompanied by altered energetic profiles. However, there are several limitations to our study. It is not known if these metabolic adaptations are integral to the SIRPα signaling axis and are required for effector rather than suppressive functions. Further investigation would be required to thoroughly assess how cellular energetics influences MDSC function. Although an increased infiltration of CD8 T cells was detected within anti-SIRPα-treated tumors, we did not evaluate their activation status or localization within tumors. Both would provide valuable information linking their presence with therapeutic impairment of tumor growth and for assessing efficacy of combination therapies with immune checkpoint blockade on T cell responses. While we implemented both cellular and peptide-based approaches to investigate functional outputs of CD47-SIRPα signaling, there remains a possibility that different sources of CD47 stand to differentially impact myeloid cell behavior. In addition, the efficacy of SIRPα on myeloid cell function was analyzed in only one murine model for each disease modality. Testing SIRPα blockade in additional murine models, particularly genetically engineered tumor models, would further support our findings.

## STAR★Methods

### Key resources table

**Table:** 

REAGENT or RESOURCE	SOURCE	IDENTIFIER
**Antibodies**		

anti-mouse CD45, Clone 30-F11	Biolegend	Cat# 103132; RRID:AB_893340, Cat# 103149; RRID:AB_2564590
anti-mouse/human CD11b Antibody, Clone M1/70	Biolegend	Cat# 101208, RRID:AB_312791; Cat# 101243; RRID:AB_356991
anti-mouse CD11c, Clone N418	Biolegend	Cat# 117318; RRID:AB_2934090
anti-Mouse Ly-6C, Clone AL-21	BD Biosciences	Cat# 553104; RRID:AB_394628, Cat# 560596; RRID:AB_1727555
anti-mouse Ly-6G, Clone 1A8	Biolegend	Cat# 127626; RRID:AB_2561340, Cat# 127614; RRID:AB_1877163
anti-mouse CD172a (SIRPα), Clone P84	Biolegend	Cat# 144012; RRID:AB_2563549, Cat# 144014; RRID:AB_2564060
anti-mouse CD274 (B7-H1, PD-L1), Clone 10F.9G2	Biolegend	Cat# 124308; RRID:AB_2073556
anti-mouse I-A^b^ (MHC class II), Clone KH74	Biolegend	Cat# 116408; RRID:AB_313726
anti-mouse CD178 (FasL), Clone MFL3	Biolegend	Cat# 106606; RRID:AB_313278
anti-mouse CD47, Clone miap301	Biolegend	Cat# 127514; RRID:AB_2562919, Cat# 127508; RRID:AB_1134133
anti-mouse VISTA (PD-1H), Clone MIH63	Biolegend	Cat# 150204; RRID:AB_2566411
anti-mouse F4/80, Clone BM8	Biolegend	Cat# 123108; RRID:AB_893502
anti-mouse CX3CR1, Clone SA011F11	Biolegend	Cat# 149008; RRID:AB_2564492
anti-mouse/rat XCR1, Clone ZET	Biolegend	Cat# 148206; RRID:AB_2563932
Arginase 1 Monoclonal Antibody, Clone A1exF5	Thermo Fisher Scientific	Cat# 17-3697-82; RRID:AB_2734835
anti-NOS2, Clone 5CB52	Biolegend	Cat# 690902; RRID:AB_2629826
IDO Monoclonal Antibody, Clone mIDO-48	Thermo Fisher Scientific	Cat# 12-9473-82; RRID:AB_2688157
anti-mouse H-2Kb bound to SIINFEKL, Clone 25-D1.16	Biolegend	Cat# 141606; RRID:AB_11219595
anti-mouse CD140a (Pdfrα), Clone APA5	Biolegend	Cat# 135912; RRID:AB_2715973
anti-mouse CD140b (Pdfrβ), Clone APB5	Biolegend	Cat# 136006; RRID:AB_1953270
anti-mouse CD31, Clone MEC13.3	Biolegend	Cat# 102506; RRID:AB_312913
anti-mouse NK-1.1, Clone PK136	Biolegend	Cat# 108732; RRID:AB_2562561
anti-mouse CD3ϵ, Clone 145-2C11	Biolegend	Cat# 100336; RRID:AB_2562556
anti-mouse CD8a, Clone 53-5.8	Biolegend	Cat# 100750; RRID:AB_2562610
anti-mouse CD4, Clone GK1.5	Biolegend	Cat# 100422; RRID:AB_312706
FOXP3 Monoclonal Antibody, Clone FJK-16s	Thermo Fisher Scientific	Cat# 45-5773-82; RRID:AB_914351
anti-mouse CD90 (Thy-1), Clone G7	Biolegend	Cat# 105202; RRID:AB_313169
Glut1 Antibody	Novus Biologicals	Cat# NB110-39113; RRID:AB_790014
Ultra-LEAF™ Purified anti-mouse CD172a (SIRPα), Clone P84	Biolegend	Cat# 144036; RRID:AB_2832520
Ultra-LEAF™ Purified anti-mouse CD3ε, Clone 145-2C11	Biolegend	Cat# 100340; RRID:AB_2616674
Ultra-LEAF™ Purified anti-mouse CD28, Clone 37.51	Biolegend	Cat# 102116; RRID:AB_2810333
Ultra-LEAF™ Purified Rat IgG1, κ Isotype Ctrl, Clone RTK2971	Biolegend	Cat# 400432; RRID:AB_11150772
CD11b Monoclonal Antibody, Clone M1/70	Thermo Fisher Scientific	Cat# 13-0112-82; RRID:AB_466359
Chicken anti-Rat IgG (H+L) Cross-Adsorbed Secondary Antibody, Alexa Fluor™ 594	Thermo Fisher Scientific	Cat# A-21471; RRID:AB_2535874
Chicken anti-Rabbit IgG (H+L) Cross-Adsorbed Secondary Antibody, Alexa Fluor™ 647	Thermo Fisher Scientific	Cat# A-21443; RRID:AB_2535861

**Chemicals, peptides, and recombinant proteins**		

Dulbecco Modified Eagle’s Medium	Thermo Fisher Scientific-Gibco	Cat# 41966029
Dulbecco Modified Eagle’s Medium, no glucose	Thermo Fisher Scientific-Gibco	Cat# 11966025
RPMI-1640 Medium	Thermo Fisher Scientific-Gibco	Cat# 11875093
IMDM	Thermo Fisher Scientific-Gibco	Cat# 12440053
Fetal bovine serum	Thermo Fisher Scientific-Gibco	Cat# 16000044
Bovine serum albumin	Sigma-Aldrich	Cat# 9048-46-8
Donkey and chicken serum	Avantar/VWR	Cat# S2170-100; Cat# 9006-59-1
PBS, pH7.4	Thermo Fisher Scientific-Gibco	Cat# 10010023
HEPES (1M)	Thermo Fisher Scientific-Gibco	Cat# 15630056
2-Mercaptoethanol	Sigma-Aldrich	Cat# M7522
Penicillin-Streptomycin (10,000 U/mL)	Thermo Fisher Scientific-Gibco	Cat# 15140122
MycoAlert® Mycoplasma Detection Kit	Lonza	Cat# LT07-318
Cell Dissociation Buffer Enzyme-Free PBS-based	Thermo Fisher Scientific-Gibco	Cat# 13151-014
Red Blood Cell Lysis Buffer	Home made	155mM NH4Cl, 12mM NaHCO3,0.1mM EDTA in ddH2O
MACS buffer	Home made	0.5% BSA and 2mM EDTA in PBS
Anti-Biotin MicroBeads	Miltenyi Biotec	Cat# 130-090-485
SCA-1-Biotin-Antibody	Miltenyi Biotec	Cat# 130-126-949
CD11b Antibody, anti-human/mouse	Miltenyi Biotec	Cat# 130-113-233
Ly-6C Antibody, anti-mouse, Biotin, REAfinity™	Miltenyi Biotec	Cat# 130-111-776
Pan T cell Isolation Kit II	Miltenyi Biotec	Cat# 130-095-130
eBioscience Foxp3 / Transcription Factor Staining Buffer Set	Invitrogen	Cat# 00- 5523
Collagenase A and D	Roche	Cat# 50-100-3278; Cat# 50-100-3282
DAPI (4',6-Diamidino-2-Phenylindole, Dihydrochloride)	Thermo Fisher Scientific	Cat# D1306
LIVE/DEAD™ Fixable Violet Dead Cell Stain Kit	Thermo Fisher Scientific	Cat# L34963
SlowFade™ Gold Antifade Mountant	Thermo Fisher Scientific	Cat# S36936
Acetone and Methanol	Thermo Fisher Scientific	Cat# 177170010; Cat# L13255.0F
Recombinant Murine GM-CSF	PeproTech	Cat# 315-03
Recombinant mouse CD47 protein (Active)	Abcam	Cat# ab231160
CellTrace™ Far Red Cell Proliferation Kit	Thermo Fisher Scientific	Cat# C34564
CellTrace™ CFSE Cell Proliferation Kit	Thermo Fisher Scientific	Cat# C34554
2′,7′-Dichlorodihydrofluorescein diacetate (DCFDA)	Sigma-Aldrich	Cat# D6883
DQ™ Ovalbumin	Thermo Fisher Scientific	Cat# D-12053
Streptavidin, Alexa Fluor™ 647 Conjugate	Thermo Fisher Scientific	Cat# S32357; N/A
2-NBDG (2-(N-(7-Nitrobenz-2-oxa-1,3-diazol-4-yl)Amino)-2-Deoxyglucose)	Thermo Fisher Scientific	Cat# N13195

**Critical commercial assays**		

ATP Assay Kit	Sigma-Aldrich	Cat# 119107
NAD/NADH Quantitation Kit	Sigma-Aldrich	Cat# MAK037

**Experimental models: Cell lines**		

B16-F10	ATCC	Cat# CRL-6475
E0771	CH3 BioSystems	Cat# 94A001
mM1	Prof. D Tuveson	N/A

**Experimental models: Organisms/strains**		

C57BL/6J	The Jackson Laboratory	Strain #:000664; RRID: IMSR_JAX:000664

**Softwareandalgorithms**		

FlowJo v10	BD Biosciences	N/A
Prism 9	GraphPad	N/A
ZEISS ZEN lite	Zeiss	N/A

**Other**		

Murine melanoma public study – Davidson et al.	Array Express	E-MTAB-7427

### Resource availability

#### Lead contact

Further information and requests for resources and reagents should be directed to and will be fulfilled by the lead contact Jacqueline D. Shields (jacqueline.shields@nottingham.ac.uk).

#### Materials availability

Key resources table including details of key reagents and cell lines used are available in the key resources table. Any unique reagents generated in this study are available from the lead contact with a completed Materials Transfer Agreement. Datasets are listed in the key resources table.

#### Data and code availability

• This paper analyses existing, publicly available data. The accession numbers for which are listed in STAR Methods and key resources table.
• This paper does not report original code.
• Any additional information can be obtained from the lead contact upon request.

### Experimental model and subject participant details

#### Mice

Wild-type C57BL/6 mice were obtained from the in-house breeding core within the MRC ARES facility. Adult female mice aged between 8 to 12 weeks of age were used for experiments. Animals were socially housed in individually ventilated cages with enrichment. All experiments were performed after review and approval by MRC Laboratory of Molecular Biology Animal and Ethical Review Board (AWERB) and approved by the Home Office in accordance with the Animals (Scientific Procedures) Act 1986 and ARRIVE guidelines. Non-invasive tumour measurements and intraperitoneal (I.P.) drug injections were performed by trained animal technicians at ARES, who, where possible, were blinded to experimental groups.

### Method details

#### B16.F10 murine melanoma model

B16.F10, B16.F10 overexpressing ovalbumin (OVA) or B16.F10-GFP cells were passaged following standard protocol and re-suspended at a density of 2.5x10^5^ in 50μL of sterile saline for injection. Cells were injected subcutaneously into the right shoulder. Mice were sacrificed 5-, 9- or 11-days post tumour induction by exposure to carbon dioxide, followed by cervical dislocation or exsanguination by cardiac puncture (when blood samples were required). For anti-SIRPα treatment, mice received 125μg of Ultra-LEAF™ Purified anti-mouse CD172a (SIRPα; 144037, Biolegend) Antibody or a rat IgG1 isotype control (400427, Biolegend) by intraperitoneal injection. The first dose was administered once tumours reached 3mm in size (normally at Day 5 post-tumour induction) followed by a second dose on Day 8 post-tumour induction. Three days later, mice were sacrificed and the tumours and blood were harvested for analysis. To measure phagocytosis *in vivo*, mice were inoculated with 2.5x10^5^ B16.F10-GFP cells and treated with or without anti-SIRPα as described above. For assays measuring the antigen presentation capacity of MDSCs, mice received 4x10^5^ B16.F10-OVA cells and were treated with anti-SIRPα as above. However, for these experiments, mice were sacrificed on Day 9 rather than Day 11 post-tumour induction for analysis. For pancreatic cancer syngeneic tumours, 1x10^6^ mM1 pancreatic cancer cells, derived from a murine model of pancreatic adenocarcinoma generated on a C57BL/6 background (kindly gifted by Professor Dave Tuveson, CSHL), were injected subcutaneously into the flank of C57BL/6 mice and treated as above, sacrificing on Day 11 post-tumour induction. For orthotopic breast tumours, we injected E0771 cells (CH3 BIOSYSTEMS) at a density of 2.5x10^5^ in 50μL into the mammary fat pad of C57BL/6 mice. Mice were treated with anti-SIRPα on days 10 and 13 and were sacrificed on day 16.

#### Processing of tumour and blood

Resected tumours were mechanically dissociated using a blade and digested in 1mg/ml collagenase D (Roche), 1mg/ml collagenase A (Roche) and 0.4mg/ml DNase (Roche) in PBS, at 37°C for 45min before collagenase activity was neutralized with 5mM EDTA. Digested tumours were then passed through 70μm cell strainers (Falcon) and washed. Single cells suspensions were pelleted at 300g for 5min, resuspended in PBS and seeded into a round-bottomed 96-well plates (Corning). Blood samples obtained from cardiac puncture were collected in K_2_EDTA tubes; Samples were incubated in 5mL of red blood cell lysis buffer (RBC Lysis Buffer; 150mM NH4Cl, 1mM KHCO3, 0.1mM EDTA in dH2O) at room temperature (RT) for 5min and then washed with 10X PBS. All samples were then stained for flow cytometry.

#### Generation of tumour conditioned media

For tumour cell conditioned medium (TCM), B16.F10 cells were grown until 60-70% confluent in Dulbecco’s Modified Eagle Medium (DMEM, Life Technologies) containing 10% Foetal Bovine Serum (FBS) and 1% penicillin/streptomycin (P/S). Medium was collected 24h later and centrifuged at 600g for 10min to remove cellular debris. Media was collected, snap frozen and stored at -80 °c.

#### Isolation of murine cells from bone marrow and spleen

Femurs and tibias were flushed from C57BL/6 mice with PBS. Cells were resuspended to obtain a single cell suspension free of bone debris. After washing, RBCs were lysed. For cell isolation from the spleen, the spleen was removed from C57BL/6 mice and disrupted using a 25-gauge needle and then passed through a 70μm strainer using a 1ml syringe plunger, before RBCs were lysed as described above.

#### *In vitro* differentiation of HSCs into MDSCs

HSCs were isolated from bone marrow using Magnetic-activated cell sorting (MACS – as described below) and half of the cells were resuspended in RPMI, supplemented with 20ng/mL GM-CSF (Peprotech, Cat: 315-03) and the other half was resuspended in a 1:1 mix of TCM and RPMI, supplemented with 20ng/mL GM-CSF. Cells were seeded at a density of 2.5x10^5^ cells and matured for 5 days with media changes performed daily. The MDSC were subsequently harvested by gentle pipetting and isolated as described below.

#### Magnetic-activated cell sorting to isolate immune populations

Sca-1^+^ HSCs were isolated from bone marrow-derived cell suspensions in MACS Buffer (0.5% v/v Bovine Serum Albumin [BSA] and 2mM EDTA in PBS) as per manufactures guidelines. For T cell isolation from spleen, cells were incubated with the Pan T cell Isolation Kit II (Miltenyi Biotec, 130-095-130) according to manufacturer’s instructions. Columns were then washed with MACS buffer and unlabelled CD3^+^ T cells were collected in the flow through.

For isolation of MDSCs and moDCs after *in vitro* differentiation from HSCs, cells were gently resuspended and collected to limit the presence of highly differentiated adherent cells. M-MDSCs and moDCs expressing CD11b and Ly6C were harvested using two different MACs kits, one to isolate CD11b+ cells (Miltenyi Biotec Cat: 130-113-233) and subsequently Ly6C^+^ cells (Miltenyi Biotec Cat: 130-111-776) as per the manufacturer's instructions. For isolation of CD11b^+^Ly6C^+^ cells from *in vivo* tumours, tumour tissue was processed until a single-cell suspension was obtained as described above, and MACS sorted in the same manner as *in vitro* cultures. In all cases, flow cytometry was performed to confirm the purity of MACS sorted HSCs, moDCs and M-MDSCs. Viable cells were counted using a hemacytometer and re-suspended at the desired concentration for *in vitro* assays.

#### *In vitro* CD47 active protein treatment on myeloid cell phenotype and function

The recombinant mouse CD47 protein (Active) (cat: ab231160) was reconstituted to 5μg/ml in PBS. Then, 50ul of solution was used to coat the wells of a 96 well, non-pyrogenic polystyrene flat or round (for T cell proliferation assay) bottom plate. Plates were sealed with parafilm and kept overnight at 4°C to allow even coating. Plates were then washed with PBS. After HSC differentiation *in vitro* in the presence of GM-CSF or GM-CSF supplemented with TCM, differentiated MDSCs were added to the plate and incubated for 2 days at 37°C and 5% CO_2_. Cells were then washed and analysed by flow cytometry.

#### SIRPα blockade on myeloid cell phenotype and function

The Ultra-LEAF™ Purified anti-mouse CD172a (SIRPα) Antibody (Cat: 144037) was prepared in the appropriate media and incubated with myeloid cells on ice for 30min prior to incubation in wells coated with active CD47. The antibody was used at the concentration of 115nM for treatment of tumours *in vivo* and used at 1nM or titrated 1:10 from 200nM for assays measuring the phagocytic capacity of the *in vitro* differentiated cells.

#### Cell labelling

For experiments assessing T cell proliferation, T cells harvested from spleen were stained with Cell-Trace CFSE as previously described (Thermo, Cat: C34554). For experiments assessing the phagocytic capacity of *in vitro* differentiated MDSCs, B16.F10 and CAFs cells were stained with Cell-Trace Far red as per the manufacturer's instructions (Thermo, Cat: C345664). Cells were re-suspended at a density of 0.5–10 x 10^6^ cells/ml in 1 mL of media. 5mM CFSE or Cell-Trace Far red, was added and cells were gently mixed and incubated for 7min, at RT. Cells were washed and resuspended in IMDM + 5% FCS + 0.5ul of β-mercaptoethanol (Sigma, Cat: M7522) for T cells, or RPMI + 10% FCS + P/S for B16.F10 and CAFs, for 20min at 37°C and 5% CO_2_ to allow the cells to recover.

#### T cell proliferation assay

96 well flat-bottomed plates were coated with 2.5μg/mL LEAF™ anti-mouse CD3e antibody (Biolegend, Clone: 145-2C11, Cat:14-0031) and incubated for 2h at 37°C. Excess antibody was washed off. CFSE-stained T cells were re-suspended in IMDM + 5% FCS + 0.5ul of β-mercaptoethanol (Sigma, Cat: M7522) + P/S supplemented with 1μg/mL of soluble anti-CD28 antibody (Biolegend, Clone 37.51, Cat: 16-0281) and seeded on the coated 96 well plate at a density of 2 x 10^5^ T cells per well. T cells were stimulated for 24h at 37°C and 5% CO_2_. T cells were then harvested and co-cultured with MDSCs isolated from tumours or differentiated from HSCs *in vitro* at a ratio of 1:4 myeloid cells:T cells. Co-cultures were incubated at 37°C and 5% CO_2_ for 48h with media replenishments performed after 24h. Samples were then prepared for flow cytometry analysis.

#### Detection of reactive oxygen species (ROS)

Ly6C^+^ cells were isolated from *in vitro* moDC and M-MDSC cultures and plated at 6x10^4^ cells per well in a 96 nonpyrogenic flat bottom plate where some wells were coated with active CD47 protein. In certain conditions, cells were pre-treated with anti-SIRPα blocking antibody as described above. The cells were incubated for 4h in GM-CSF alone, GM-CSF-TCM, GM-CSF-TCM-activeCD47 or anti-SIRPα-GM-CSF-TCM-activeCD47. After incubation, cells were washed and treated with 10μM 2′,7′-Dichlorodihydrofluorescein diacetate (DCFDA; Sigma-Aldrich) in basal DMEM for 25min. Immediately after, cells were washed twice with PBS and transferred to ice. Then, they were resuspended in PBS with Live/Dead Fixable Violet (Thermo, Cat: 62248) viability dye, diluted 1:1000, for 3min, to label dead cells. Samples were Immediately washed once with PBS and run on an LSR Fortessa cell analyzer (BD, Biosciences) to measure DCFDA levels as a readout for intracellular ROS.

#### Detection of metabolites in moDCs and M-MDSCs

For measurements of glucose uptake and GLUT-1 expression, we depleted glucose in myeloid cells for 4h and then treated with 200μM of a fluorescently-labeled deoxyglucose analog (2-NBDG; Invitrogen, cat: N13195) for another 20min at 37°C and 5% CO_2_. In the meantime, the GLUT1 (Novus Biologicals, Cat: NB110-39113) antibody was preincubated with an Alexa-fluor-647 chicken anti-rabbit APC (used at 35nM; Life technology, Cat: A21443). Immediately after, cells were washed and rapidly stained with the GLUT1 antibody complex at 4°C and analysed by flow cytometry. For ATP detection, cells were analysed using an ATP detection kit (Merck, Cat: 119107), according to the manufacturer’s protocol. To measure NADH, a hexokinase colorimetric assay was used. The activity of hexokinase in cellular lysates was analysed by measuring the NADH production over time, according to the manufacturer’s protocol (Sigma–Aldrich, Cat: MAK037).

#### Phagocytosis assays

To generate CD47-expressing cell debris for phagocytosis assays, B16.F10 (expressing little CD47) cells and CAFs (expressing high levels of CD47) were stained using Cell Trace Far red (Thermo, Cat: C345664) and resuspended to a concentration of 3x10^7^ cells/ml in a 1:1 mix of TCM and RPMI + 10% FCS + P/S media supplemented with 20ng/mL GM-CSF (Peprotech, Cat: 315-03). Half of the labelled cells were killed to generate cell debris by heat induction in a thermomixer for 5 minutes at 98°C. The dead cell debris was chilled in ice and mixed again with the remaining live cells.

Different myeloid populations obtained from differentiated HSCs were seeded at 5x10^4^ cells per well in a 96 nonpyrogenic flat bottom well plate and kept overnight in a 1:1 mix of TCM and RPMI + 10% FCS + P/S supplemented with 20ng/mL GM-CSF (Peprotech, Cat: 315-03). After treating with anti-SIRPα blocking antibody, cells were added to CD47 coated plates. Then, 50μl cell suspension containing 7.5x10^4^ live and 7.5x10^4^ dead labelled CD47 high or low cells was added to the wells containing the different myeloid populations and incubated for 4h at 37°C and 5% CO_2_. Subsequently, co-cultures were washed with PBS, put on ice to block further phagocytosis and stained for flow cytometry to detect the degree of phagocytosis by the myeloid cells based on Cell Trace Far Red levels detected in the myeloid cells.

#### OVA processing

Myeloid cells exposed to control media, SIRPα blockade and/or CD47 coating were pulsed with DQ-Ovalbumin (Cat: D-12053, Thermo) at 100μg/ml for 10min at 37°C and then washed 3 times with ice cold PBS containing 5% FBS. Then, the media was exchanged and samples were incubated for a further 35min. Cells were then washed and transferred to ice for flow cytometry staining.

#### Flow cytometry staining

Samples were resuspended in PBS with Live/Dead Fixable Violet (Thermo, Cat: 62248) viability dye, diluted 1:1000, for 15min. After washing, samples were incubated with fluorophore-conjugated primary antibodies prepared at 1:300 dilution in FACS buffer (0.5% BSA in PBS) and mixed 1:1 with Fc block (generated in house from a rat 2.4G2 hybridoma cell line)), for 40min, at 4°C. After surface staining, and if intracellular epitope detection was required, samples were fixed, permeabilised and stained in accordance with the FoxP3/ Transcription Factor Staining Kit (eBioscience, Cat: 00- 5523). Briefly, cells were incubated with fluorophore-conjugated primary antibodies, diluted 1:300 in permeabilization buffer, for 30min at RT. After washing, samples were run on an LSR Fortessa cell analyzer (BD Biosciences) and analysed using FlowJo version 10. (FlowJo, BD Biosciences).

#### Immunofluorescence staining

10μm frozen tissue sections were air dried and fixed in a 1:1 mix of acetone and methanol, for 2min at -20°C. Next, sections were washed in PBS for 10min before incubation in blocking solution containing 10% chicken or donkey serum and 2% BSA for 1h, at RT. The sections were then placed in a humidified chamber and incubated with unconjugated primary antibodies against SIRPα (1:50, P84 Biolegend), CD11b (1:100, biotin conjugated M1/70 eBiosciences) and fluorescently conjugated Ly6C (1:50, AL-21 Biolegend) diluted in blocking buffer, overnight at 4°C. Following 3 x 5min washes in PBST (PBS with 0.1% Tween), sections were incubated with 1:300 Chicken anti-Rat Conjugated AF594 (A21471; for SIRPα) and Streptavidin conjugated AF 647 (S32357; for CD11b, both from Life Technologies) for 1h, at RT. Sections were then counterstained with 1μg/ml of 4',6-diamidino-2-phenylindole (DAPI, Thermo, D1306), for 10min, and mounted onto 22 x 50 mm glass coverslips with SlowFade Gold Antifade Mountant (Life Technologies; Cat: S36936). Sections were imaged on a Zeiss 880 laser scanning confocal microscope using a 40x oil objective (ZEISS).

#### Analysis of public datasets

To evaluate expression of CD47 and SIRPα patterns within the murine melanoma microenvironment, single cell data from Davidson et al.^58^ was accessed online from http://www.teichlab.org/data/. The raw sequencing data is also available from ArrayExpress: E-MTAB-7427 (deposited by authors).

### Quantification and statistical analysis

To evaluate statistical significance between two samples a t-test was performed. For multiple comparisons, one way or two-way ANOVA were employed with a Dunnett, Šidák or Tukey post- hoc test depending on the pairwise comparisons being performed. Data are expressed as mean ± SEM, where a different cell isolate and batch of TCM was used for each experiment and was therefore considered a different biological sample. Data were analysed using Graphpad Prism 9 Software packages. Some figure images were created with assistance from Biorender.
