# Supplementary material for: Disruption of CD47-SIRPα signaling restores inflammatory function in tumor-associated myeloid-derived suppressor cells
Source: iScience. 2024 Mar 20;27(4):109546. doi: 10.1016/j.isci.2024.109546 (PMC10993187; doi:10.1016/j.isci.2024.109546)
Supplement: Document S1. Figures S1–S5 [file mmc1.pdf]

iScience, Volume *27*

Supplemental information

Disruption of CD47-SIRPα signaling restores inflammatory function in tumor-associated myeloid-derived suppressor cells

Carlo Zimarino, William Moody, Sarah E. Davidson, Hafsa Munir, and Jacqueline D. Shields

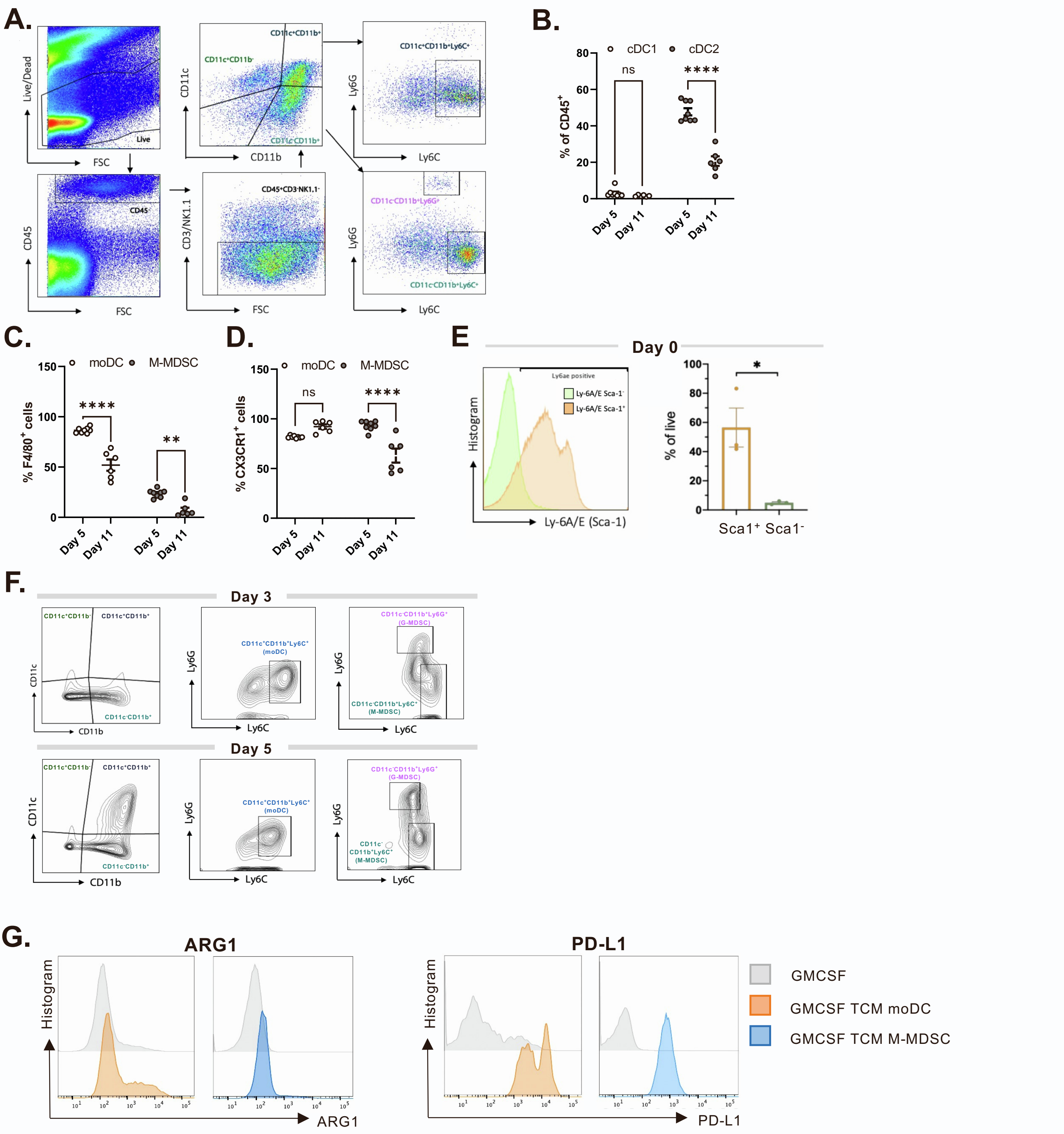

Supplementary Figure 1, Related to Figure 1-2. Characterisation of the myeloid landscape of B16-F10 tumours and in vitro. (A) Representative FACS plots of tumour at day 11 post-induction showing the gating strategy for identifying MDSCs. Live cells were gated followed by $CD3^+$ and $NK1.1^+$ cells excluded. Within $CD11c^+CD11b^+$ and $CD11c^-CD11b^+$ gates, $CD11c^+$ (moDC), $Ly6G^+$ (G-MDSC) and $Ly6C^+$ (M-MDSC) cells were identified. **(B)** Quantification of $CD11c^+CD11b^-XCR1^+$ (cDC1) and $CD11c^+CD11b^+Ly6C^-$ (cDC2) cells at day 5 and 11 post-tumour induction. Quantification of **(C)** F4/80 and **(D)** CX3CR1 expression by moDCs, M-MDSCs at day 5 and 11 post-tumour induction. **(E)** Differentiation of Sca1+ HSC *in vitro* to model myeloid landscape; Quantification of Sca-1 expression of HSC immediately post isolation (Day 0), and representative flow cytometry plot. **(F)** Representative flow cytometry plots from 8 independent experiments depicting gradual myeloid maturation by distribution of CD11b and C after 3 and 5 days of culture in GM-CSF. **(G)** Representative flow cytometry histograms showing ARG1 and PD-L1 expression in MoDC and M-MDSC treated with GM-CSF or GM-CSF+TCM. Data are mean ± SEM; * = $p < 0.05$, ** = $p < 0.01$, *** = $p < 0.001$, **** = $p<0.0001$. (B-D) Two-way ANOVA with a Sidak's multiple comparison post hoc test. (B) For day 5, n=4 mice and for day 11, n=3 mice performed in duplicate. (C-D) n=4 for both time points from two (day 5) and three (day 11) independent experiments.

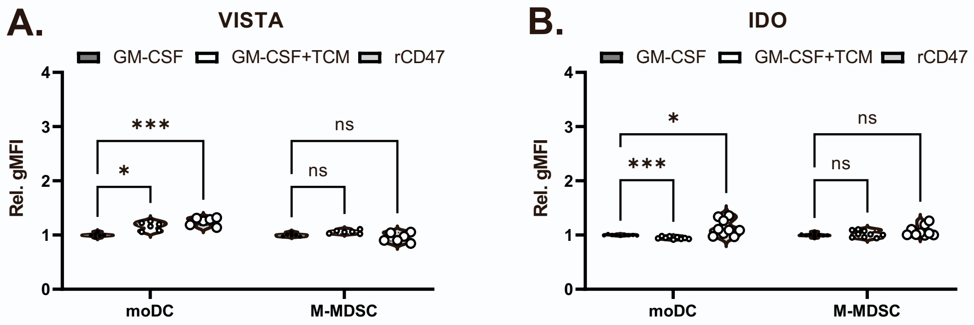

Supplementary Figure 2, Related to Figure 2. Immunomodulatory molecules expressed by MDSCs. Quantification of immune modulatory marker expression by flow cytometry shown as MFI geometric mean for **(A)** VISTA and **(B)** IDO expression in each myeloid cluster (expressed as relative gMFI normalized to GM-CSF). Data are mean ± SEM; * = p < 0.05, ** = p < 0.01, *** = p < 0.001, **** = p<0.0001. (A-B) Two-way ANOVA with a Šidák's multiple comparison post hoc test. (A-B) n=3.

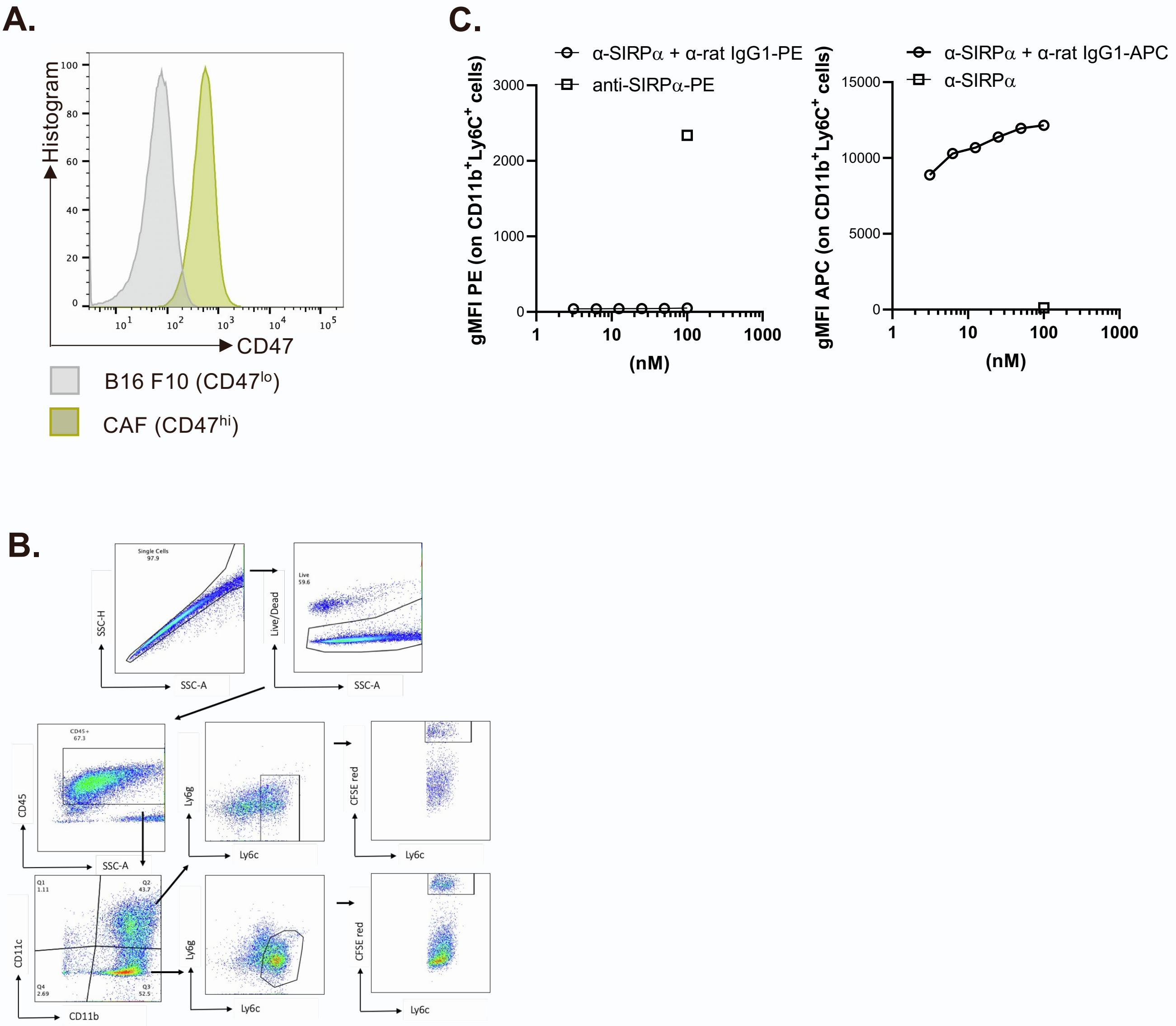

Supplementary Figure 3, Related to Figure 5. CD47-SIRPα modulation of phagocytosis and antibody binding. (A) Representative flow cytometry histogram showing CD47 expression on B16 (CD47lo and CAF CD47hi) cells. (B) Gating strategy used to analyse uptake of CFSE red stained cell debris by moDC and M-MDSC. (C) Competition assay showing occupation of SIRPα eptipoes by anti-SIRPα antibody. Left panel: Quantification of gMFI signal detected for fluorophore conjugated anti-SIRPα antibody after epitope blockade by Ultra LEAF- SIRPα antibody. Right panel: Quantification of gMFI signal detected for Ultra-LEAF antibody detected by fluorophore conjugated Rat IgG. Data are mean ± SEM; ** = p < 0.01, *** = p < 0.001, **** = p<0.0001. (C) n=3 independent assays performed in triplicate.

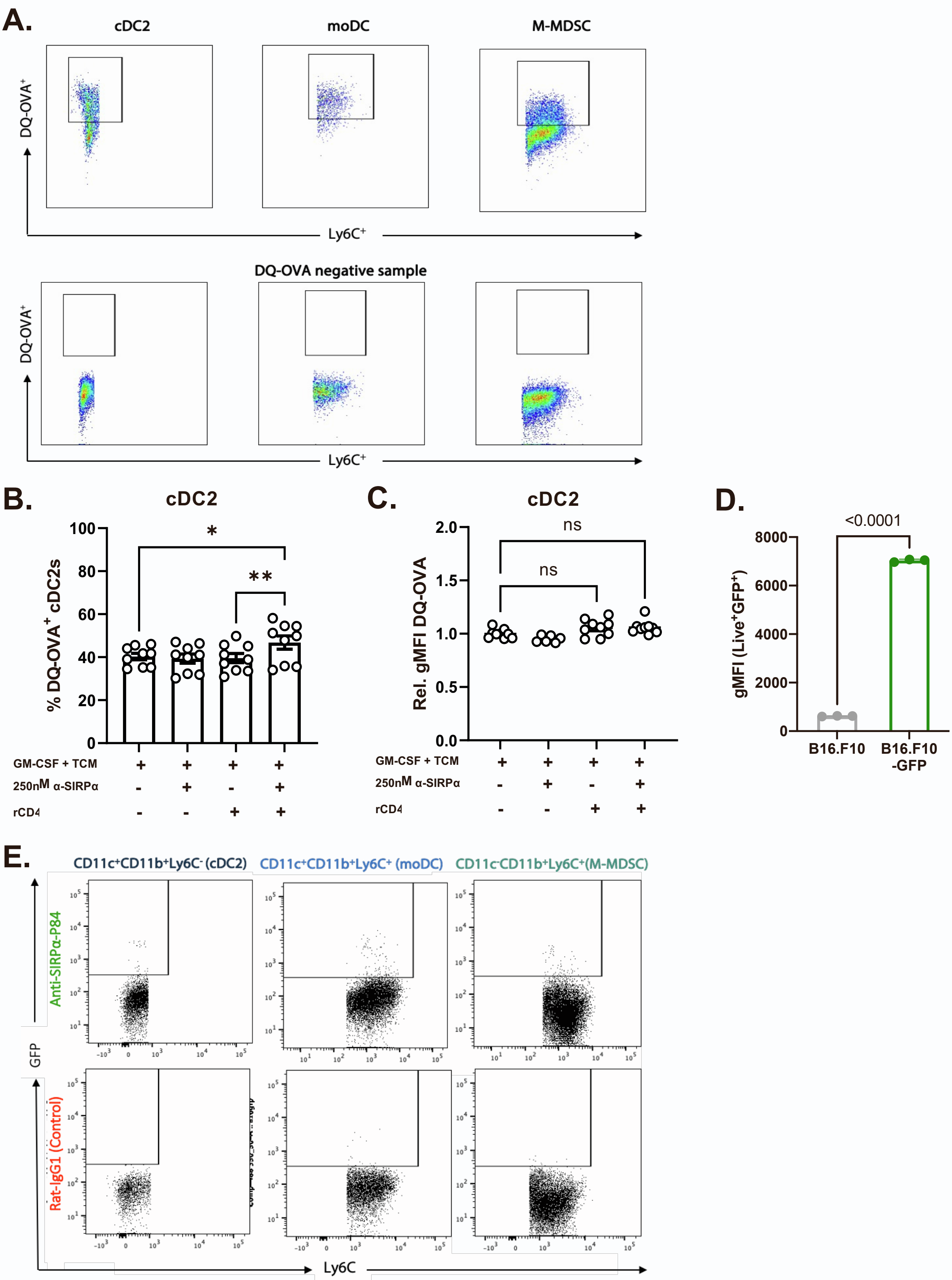

Supplementary Figure 4, Related to Figure 7. SIRPα blockade facilitates phagocytosis, antigen processing and presentation by myeloid cells. (A) Representative flow cytometry plots showing signal detected for processed DQ-OVA vs DQ-OVA negative controls in cDC2, moDC and M-MDSCs. (B) Quantification of the percentage of GM-CSF supplemented TCM treated cDC2s that uptake and proteolytic process DQ-OVA antigen with or without CD47 stimulation in the presence or absence of anti-SIRPα. (C) Quantification of the relative levels of DQ-OVA processed by cDC2s (gMFI). Data normalised to GM-CSF-TCM condition. (D) Quantification of gMFI of the GFP fluorescent protein in a stably transduced B16-F10 melanoma cell line. (E) Representative FACS showing uptake of GFP-labelled tumour material by cDC2, moDC and M-MDSC cells between anti-SIRPα injected mice and control. (Data are mean ± SEM; * = $p < 0.05$, ** = $p < 0.01$. (B-C) One-way ANOVA with Tukey's multiple comparisons and Dunnett's post hoc test, respectively. (D) Unpaired t test. (B-C) n=4 mice for each of two independent experiments.

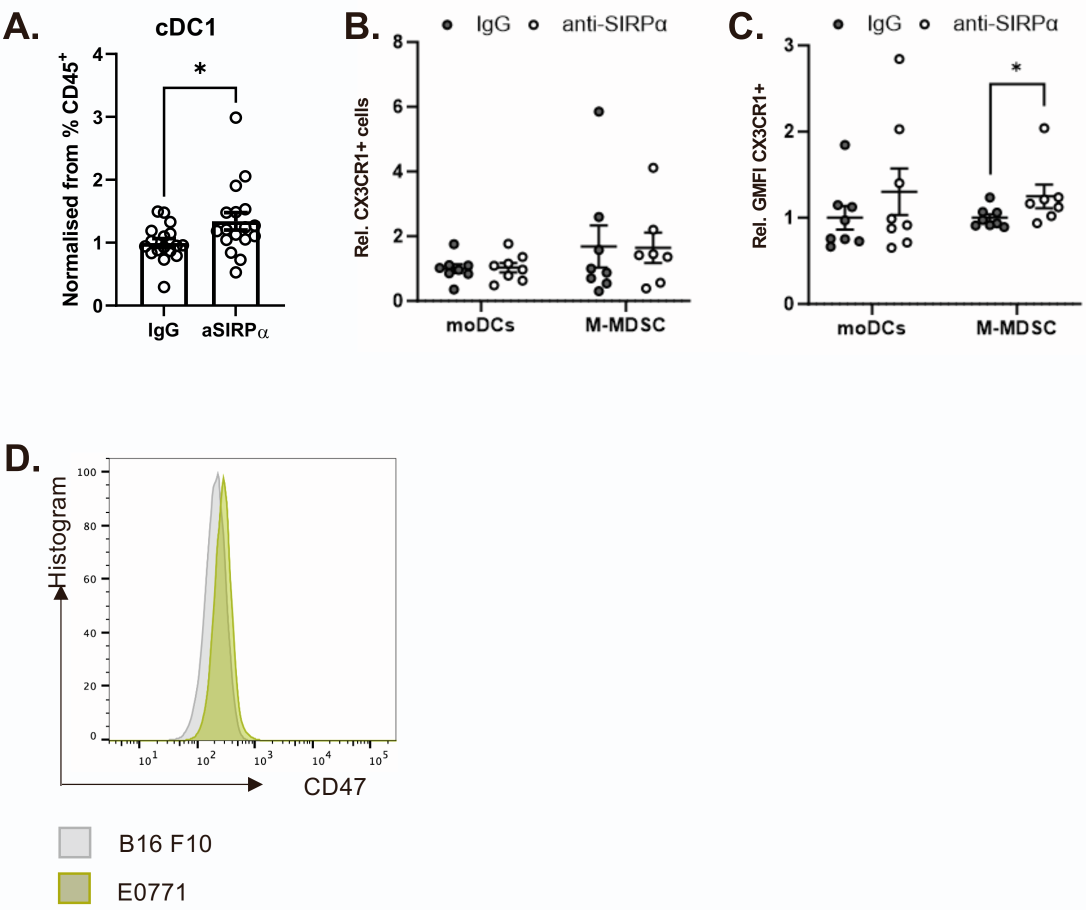

Supplementary Figure 5, Related to Figure 8. Sirpα blockade remodels myeloid composition in vivo. (A) Quantification of tumour infiltrating cDC1s (normalised to the percentage of CD45+ cells) after treatment with anti-SIRPα or isotype control. (B) Quantification of the proportion of CX3CR1+ moDCs and M-MDSC after treatment with anti-SIRPα or isotype control. Data were normalised to the rat-IgG1 controls. (C) Quantification of the expression of CX3CR1 on moDCs and M-MDSC after treatment with anti-SIRPα or isotype control. Data were normalised to the rat-IgG1 controls. (D) Representative flow cytometry plot showing comparable CD47 expression in B16F10 and E0771 breast cancer cells. Data are mean ± SEM; * = $p < 0.05$, ** = $p < 0.01$. (A-C) Unpaired t test. (A) n=3 independent experiments each with n=3 per group. (B-C) n=4 for each group from 2 independent experiments.
